# HS‐FET‐GC/MS‐Method Development and Validation for Analysis of 45 Terpenes—Creating a Complementary Tool for Comprehensive Profiling of Cannabis Flowers in Forensics

**DOI:** 10.1002/dta.3966

**Published:** 2025-11-20

**Authors:** Marica Hundertmark, Tanja Germerott, Cora Wunder

**Affiliations:** ^1^ Department of Forensic Toxicology, Institute of Legal Medicine University Medical Center Mainz Mainz Germany

**Keywords:** cannabis, forensic toxicology, full evaporation technique, strains, terpenes

## Abstract

*Cannabis sativa* is one of the oldest and most versatile plants with many facets ranging from intoxicant to medicine. Legalisation of medicinal cannabis leads to an increasing complexity of specific forensic questions to distinguish between recreational and medicinal use, for example, in context with participation in road traffic. Hence, there is a recent interest in finding objective markers that enable the differentiability of cannabis flowers. Terpenes, volatile hydrocarbons with a modular construction principle of isoprene subunits, are currently suggested as a second substance class alongside phytocannabinoids for the classification of cannabis material. A headspace full evaporation technique gas chromatography mass spectrometry (HS‐FET‐GC/MS) methodology was successfully validated according to forensic guidelines for the analysis of 45 terpenes in cannabis flowers including 16 monoterpenes, 16 monoterpenoids, 7 sesquiterpenes and 6 sesquiterpenoids. FET‐sampling was developed in detail experimentally, revealing evidence of thermal instability of higher‐boiling terpenes. Validation included selectivity, linearity of calibration (ranges 10–2000 μg/g), analytical limits (at least 6 μg/g), accuracy (bias) as well as intraday and interday precision. The use of a retention time index mixture as an internal standard and measurement in SIM‐scan mode also allows for the qualitative identification of further terpenes present in cannabis. Application to a set of cannabis strains with similar Δ^9^‐THC content demonstrated differences and similarities in their terpene profiles.

## Graphical abstract

Legalisation of medicinal cannabis leads to an increasing complexity of specific forensic questions. Terpenes, volatile hydrocarbons with modular construction principle of isoprene subunits, are suggested as second substance class alongside phytocannabinoids for classification of cannabis material. A headspace full evaporation technique gas chromatography mass spectrometry (HS‐FET‐GC/MS) methodology was developed and successfully validated according to forensic guidelines for analysis of 45 terpenes in cannabis flowers. Application to cannabis strains with similar Δ^9^‐THC‐content demonstrated differences and similarities in their terpene profiles.

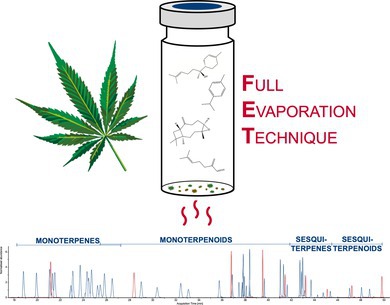

## 1 Introduction

As one of the oldest cultivated plants *Cannabis sativa* has been grown for millennia under different climatic conditions making it a particularly versatile plant [1] with many facets ranging from intoxicant to medicine. On the one hand, it remains the most commonly used drug with approx. 244 million consumers worldwide in 2023 equalling 4.6% of the global population between 15 and 64 years of age [2]. On the other hand, 64 countries have developed provisions in their national legislation or at least corresponding guidelines for the medicinal use of cannabis by 2021 [3]. In Germany, an amendment of the law in March 2017 allowed the prescription of medicinal cannabis flowers which are not approved under medicinal law [4]. Since then, a trend towards diversification of flowers with increasingly high Δ^9^‐tetrahydrocannabinol (Δ^9^‐THC) levels can be observed on the German medicinal cannabis market. While in 2017 there were only 14 products with a maximum of 22 wt‐% Δ^9^‐THC [5], currently more than 900 products containing up to 35 wt‐% Δ^9^‐THC are available [6]. Application patterns of patients reflect high‐dose inhalation of cannabis flowers with high Δ^9^‐THC contents [7], creating the impression that trends from the recreational sector overflow the medicinal cannabis market and blur the boundaries between both. Depending on national jurisdiction, the introduction of cannabis as medicine may lead to issues with other legislations, for example, drug driving regulations under traffic law [8, 9, 10]. In Germany, medicinal cannabis patients are allowed to take part in road traffic as long as they do not show signs of impairment [10]. Specific forensic questions to differentiate recreational and medicinal use may arise, which are to be addressed by an expert witness in court. From a forensic point of view, there is hence an increasing interest in finding markers that enable the differentiability of cannabis flower material [11, 12].

For this reason, it is worth developing a deeper knowledge of the numerous systems that can be found in the literature to classify the great diversity of cannabis. According to its main cannabinoid cannabis can broadly, though legally helpful, be categorised into chemotypes [13, 14]. In the context of illegal/recreational cultivation, however, unscientific descriptions are used with terms that have adopted different meanings in the vernacular than their original scientific definition was [1, 15]. Especially Δ^9^‐THC‐dominant chemotypes are often further subdivided using the terms ‘sativa’, ‘indica’ or ‘hybrid’, which are originally derived from botanical taxonomy [16]. ‘Sativa’ plants are said to have a high Δ^9^‐THC content with a sweetish aroma causing a euphoric, stimulating ‘head‐high’. ‘Indica’ plants with moderate Δ^9^‐THC levels and a sour‐biting aroma were reported to be better tolerated and to cause a ‘body‐high’ with sedative properties [1, 17]. Since the 1970s, an excessive number of roughly estimated > 700 [13] to > 8000 [18] cannabis cultivars (‘strains’) have been bred [1, 17, 19]. Since these strains mainly originate from illegality, information on genetic relationships between them is usually incomplete [20] and very few are sufficiently described to be considered real ‘cultivars’ in the botanical sense [21]. Despite the questionable scientific evidence, medicinal cannabis is still marketed today with strain names, classified as ‘sativa’, ‘indica’ or ‘hybrid’. The recording of a comprehensive chemical fingerprint has been recently proposed as an objective way, to classify medicinal cannabis flowers into ‘chemovars’ [13]. For the characterisation of chemovars, terpenes are currently used as a second substance class alongside cannabinoids [13, 19, 22, 23, 24, 25, 26].

**FIGURE 1:**
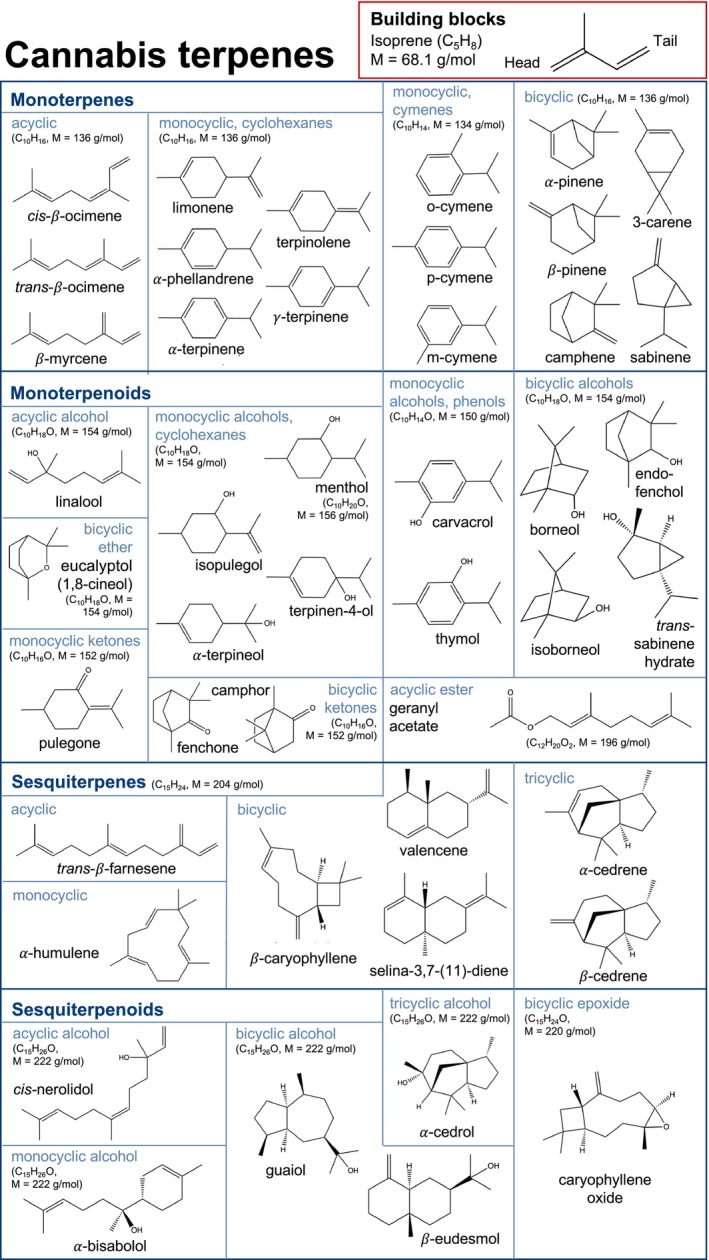
Structural formula of selected cannabis terpenes included in the analysis.

Terpenes are a particularly diverse, sometimes even referred to as the largest [27], group of plant chemicals with up to 30,000 characterised substances [28]. The volatile hydrocarbons follow a modular construction principle of isoprene subunits; for example, representatives with two isoprene subunits are called monoterpenes (C_10_H_16_, M = 136 g/mol), while terpenes with three isoprene units are referred to as sesquiterpenes (C_15_H_24_, M = 204 g/mol). If they also contain oxygen, for example, in the form of alcohols, ethers, esters or epoxides, they are called terpenoids (Figure 1) [28, 29]. At least 120 terpenes have been identified in the cannabis plant so far, among them 61 monoterpenes and 51 sesquiterpenes [30], making them the second largest group of constituents after phytocannabinoids. As terpenes occur ubiquitously in the plant kingdom, their investigation in cannabis has received substantially less research interest in the past than phytocannabinoids which are specific to them [31]. However, apart from the recent increase in medicinal interest in them, for example due to possible entourage effects between phytocannabinoids and terpenes [32], terpenes have also occasionally been studied in cannabis material in the context of other backgrounds. From a forensic perspective, comprehensive analytical profiles including terpenes, were recorded as an approach to trace the geographical origin of illegally confiscated material [33, 34, 35, 36, 37, 38, 39]. As volatile terpenes contribute to the odour of cannabis, they have also been the focus of studies aimed at uncovering its characteristic smell [40, 41, 42]. In addition to genetics, terpene profiles have been investigated as an approach to an objective chemotaxonomic classification [43, 44, 45].

While analysis of cannabinoids is already well implemented in forensics by either gas chromatography mass spectrometry (GC–MS) [46] or liquid chromatography tandem mass spectrometry (LC–MS/MS) [47, 48], the additional detection of minor cannabinoids besides Δ^9^‐THC could not sufficiently answer all complex questions to differentiate between medicinal cannabis and illegal/recreational cannabis [12, 49]. The detection of terpenes as a second dimension could therefore be a helpful approach to objectively and analytically scrutinise statements regarding seized/consumed cannabis material. Due to the recent increase in research interest in terpenes, the number of scientific publications on terpene analysis in cannabis flowers, mainly using GC, has increased significantly in the past few years although the quality of the analytical approaches differs greatly. Isomerism of terpenes makes analysis challenging, including difficulties in identifying co‐elutions and unclear defined compositions of reference substances. Early studies were often performed using identification via mass spectrometry (MS) libraries without reference standards; percentage values were estimated using relative responses delivered after flame ionisation detection (FID) [50]. When quantifying using FID, often only a few standards were calibrated, and calibration functions were used as surrogates for other analytes, since similar structures exhibited similar response factors [13, 51]. Comparing different studies, discrepancies regarding major constituents and concentration ranges of individual terpenes have been noticed [52]. Since some studies also report terpenes that are missing in other studies, misidentification of isomers may be assumed [19]. From an analytical point of view, there are differences with regard to the detection method [53] as well as sample application techniques [52], including solvent extraction with subsequent liquid injection [54] or headspace‐based techniques such as static headspace (SHS) and solid phase microextraction (SPME) [55]. The full evaporation technique (FET) is a special form of SHS and has recently been proposed in literature for terpene analysis in cannabis flowers [56, 57]. Unlike conventional SHS, FET is not based on establishing an equilibrium in the vial, but rather on complete evaporation of volatile components in the sample. Usually, very small sample quantities are thermostatted at high temperatures for a short period of time. FET was developed specifically for applications where no analyte‐free matrix is available [58]. This study places a strong focus on a structured and careful development of a HS‐FET‐GC/MS methodology, its validation and exemplary application for the detection of terpenes in cannabis flowers according to forensic guidelines.

## 2 Material and Methods

### 2.1 Material

#### 2.1.1 Chemicals and Reference Material

The majority of terpenes was bought in the form of four commercial mixtures. ‘Cannabis Terpene Mixes A and B’ (2,000 μg/mL in methanol) were obtained from Merck (Darmstadt, Germany). ‘Terpenes Mega Mix #1’ (2,500 μg/mL in isopropanol) was from Restek (Bad Homburg, Germany) and ‘Terpene Mixture 1’ (2,500 μg/mL in hexane) was from Dr. Ehrestorfer (Augsburg, Germany). A list of terpenes contained in individual mixtures is provided in Table 1. Apart from these mixtures, 1 mg of selina‐3,7‐(11)‐diene was purchased from Toronto Research Chemicals (Toronto, Canada, Purity 95%) and dissolved in 100 μL methanol to prepare a stock solution of 10 mg/mL. A retention time index standard mix (Restek, Bad Homburg, Germany) containing linear alkanes between n‐heptane (C7) and n‐tritiacontane (C33) in concentrations between 100 and 200 μg/mL in hexane was used as an internal standard (ISTD, relevant components: n‐decane (C10), n‐undecane (C11), n‐dodecane (C12), n‐tridecane (C13), n‐tetradecane (C14), n‐hexadecane (C16), n‐heptadecane (C17), 100 μg/mL each and n‐pentadecane (C15), 200 μg/mL). Reference substances were stored at −20°C fulfilling the manufacturers' recommendation given in the certificate of analysis. Dilutions of commercial terpene mixtures were prepared in HPLC‐grade methanol (≥ 99.8%, Fisher chemical, Waltham, Massachusetts, USA). Samples were prepared in 20 mL headspace‐screw‐thread vials with corresponding caps with PTFE/silicone‐septa (temperature stability up to 200°C). Cannabis flowers were ground using an Ultra‐Turrax Tube Drive (IKA GmbH & CO. KG, Staufen, Germany) and weighed using an analytical balance (Sartorius, Göttingen, Germany).

**TABLE 1:** Overview of analytes, composition of commercial terpene mixtures and mass fragments.

No.	Analyte	Commercial terpene mixes				T, Q1, Q2
		1	2	3	4	
1	α‐pinene	**x**	x			**93**, 121, 136
2	camphene	**x**	x		x	**93**, 77, 121
3	sabinene	**x**				**93**, 77, 136
ISTD	DECANE					**142**, 113
4	β‐myrcene	**x**			◯	**93**, 121, 136
5	β‐pinene	**x**	x	x		**93**, 69, 77
6	α‐phellandrene	◯			**x**	**93**, 91, 136
7	3‐carene	**x**	x	x	x	121, **93**, 77
8	α‐terpinene	**x**	x			**121**, 93, 136
9	*cis*‐β‐ocimene	⦻ (28.0%)			◯ (24.6%)	**93**, 91, 79
10	m‐cymene	**x**				**119**, 134, 117
11	limonene	**x**	x	x		**68**, 93, 121
12	p‐cymene	**x**		x	x	**119**, 117, 115
13	*trans*‐β‐ocimene	⦻ (72.0%)			◯ (69.1%)	**93,** 79, 121
14	eucalyptol	**x**			x	**108**, 111, 154
15	o‐cymene	**x**				**119**, 134, 91
16	γ‐terpinene	**x**	x			**93**, 121, 79
ISTD	UNDECANE					**156**, 127
17	terpinolene	**x**		x		**93**, 121, 136
18	*trans*‐sabinene hydrate	**x**				71, **93**, 121
19	linalool	**x**		x	x	**71**, 93, 121
20	fenchone		**x**			**81**, 69, 152
ISTD	DODECANE					**170**, 127
21	fenchol	**x**	x			**81**, 80, 69
22	isopulegol	**x**				121, **81**, 80
23	camphor		**x**	x		**95**, 81, 108
24	isoborneol	x	**x**		x	**95**, 121, 110
25	terpinene‐4‐ol	**x**				111, **71**, 93
26	menthol	x	**x**		x	**81**, 95, 71
27	borneol	xx		**x**		**95**, 110, 93
28	α‐terpineol	**x**		x		**93**, 121, 81
ISTD	TRIDECANE					**184**, 127
29	pulegone		x			**81,** 69, 93
30	thymol	**x**				**135**, 150, 115
31	carvacrol	**x**				**135**, 150, 151
ISTD	TETRADECANE					**198**, 155
32	geranyl acetate		**x**		x	**69**, 93, 121
33	*trans*‐β‐farnesene	**x**			◊	**69**, 67, 81
34	α‐cedrene	x (80.0%)	**x**			**119**, 105, 204
35	β‐caryophyllene	x		**x**	x	**93**, 161, 120
36	β‐cedrene	**x** (20.0%)				**161**, 93, 120
ISTD	PENTADECANE					**212**, 169
37	α‐humulene	***○***	**x**			**93**, 80, 121
38	valencene	***○***			⦻	**161**, 175, 176
39	*cis*‐nerolidol	◊	◊	**x**	x	**69**, 93, 107
ISTD	HEXADECANE					**226**, 169
40	selina‐3,7‐(11)‐diene				Spike	**161**, 122, 204
41	guaiol	**x**				**161**, 197, 93
ISTD	HEPTADECANE					**240**, 155
42	caryophyllene oxide				x	**79**, 93, 107
43	α‐cedrol	x	**x**		x	**95**, 150, 151
44	α‐bisabolol	x	**x**			**69,** 109, 119
45	β‐eudesmol			**x**		**59**, 149, 164

*Note:* Four commercial terpene mixes (1 = Terpenes Mega Mix #1 from Restek, 2 = Cannabis Terpene Mix A from Merck, 3 = Cannabis Terpene Mix B from Merck, 4 = Terpene Mixture 1 from Dr. Ehrestorfer) were used. As some analytes occur in several terpene mixes, the mix used for calibration is highlighted by a **x** in bold. Some analytes in these mixtures were present in purities < 95% (marked by ○). If calibration had to be performed with reference substances in a purity < 95%, this was marked by ⦻. If only incomplete information on the isomer composition of the analytes was available in the certificate of analysis, this was not taken into account for quantitative purposes (marked by ◊). In case of Terpenes Mega Mix #1, borneol was included as $\;\left(-\right)$‐ and $\;\left(+\right)$‐enantiomers, which results in twice the amount when analysed achirally (marked by xx). Target (T) and qualifier (Q1, Q2) masses are listed. Usually the strongest mass fragments (bold) were chosen as a target.

### 2.2 Methods

#### 2.2.1 HS‐GC/MS Instrument

Analysis was performed on an Agilent 8890 GC coupled with an Agilent 5977C GC/MSD and a PAL3 series I autosampler to perform headspace injections with a 2,500 μL gas tight syringe. The system was equipped with an Agilent VF‐624 ms column (60 m × 0.32 mm × 1.80 μm) containing a mid‐polar (6% cyanopropyl/phenyl, 94% polydimethylsiloxane) phase.

#### 2.2.2 Method Development

**FIGURE 2:**
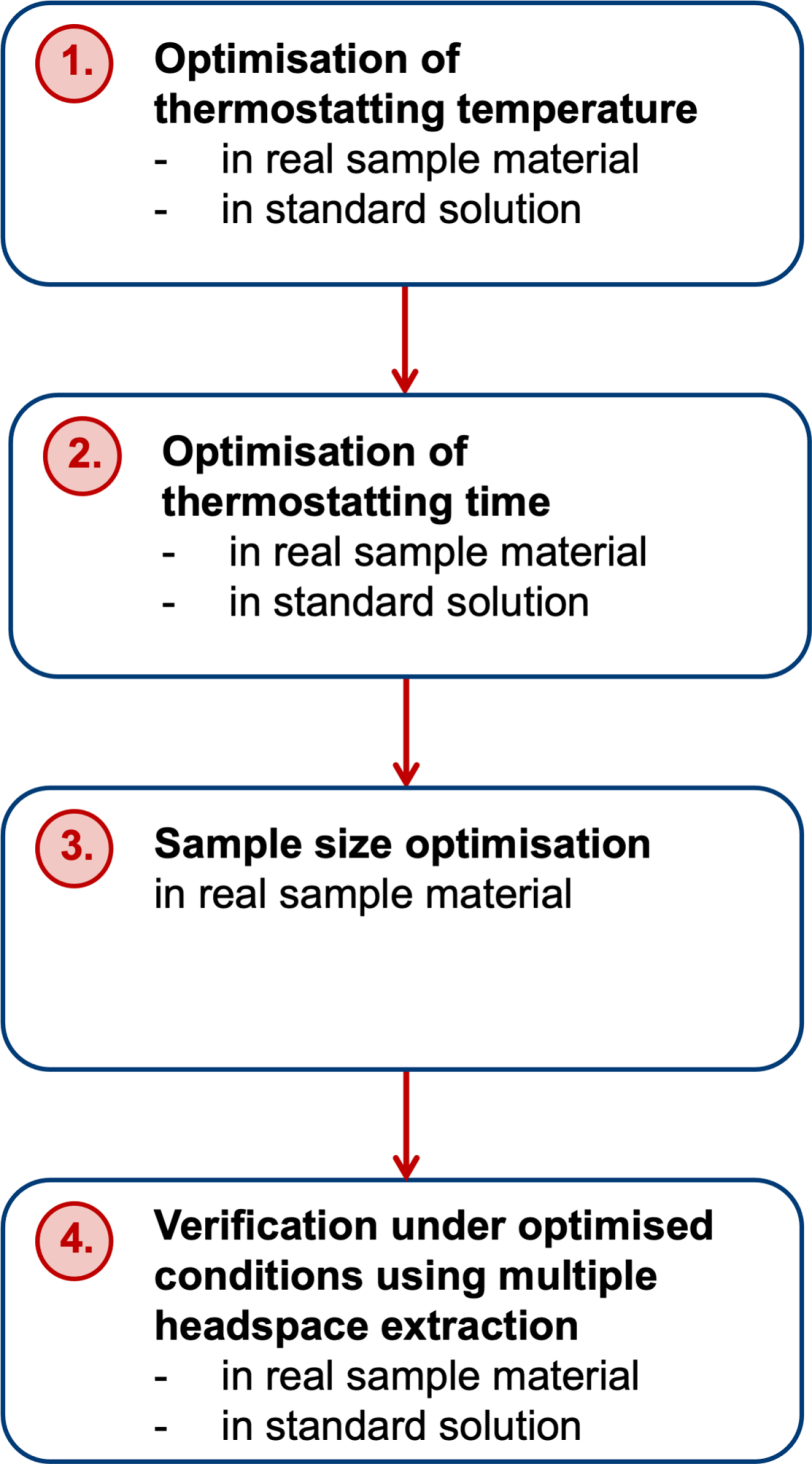
Proposed workflow for experimental verification of full evaporation.

Sample introduction was performed by FET according to Markelov and Guzowski [58]. In order to verify the condition of full evaporation, several experimental steps were carried out to optimise headspace sampling parameters (Figure 2). Firstly, approx. 5 mg of a ground cannabis flower resp. 10 μL of standard (100 μg/mL solution of Terpenes Mega Mix #1) were thermostatted for 10 min at increasing temperatures between 70°C and 170°C (10°C steps, *n* = 2 per condition) which covers the range of FET applied to cannabis flowers in literature before [56, 57, 59, 60] to determine the temperature at which no further increase in sensitivity occurs. Since complete evaporation may not necessarily appear instantaneously [61], different thermostatting times in a range between 2.5 and 40 min (5 min steps, *n* = 2 per condition) were tested at the previously optimised temperature (100°C). With the adjusted thermostatting conditions (100°C, 20 min), a sample size optimisation using increasing amounts of ground cannabis flowers in a range between 2.5 and 15 mg was conducted. To verify the condition of full evaporation under optimised conditions (100°C, 20 min, 5 mg ground cannabis material resp. 10 μL methanol standard) a multiple headspace extraction (MHE) was performed with five injections. For FET experiments samples were spiked with 2 μL ISTD.

#### 2.2.3 Final Methodological Parameters

For the final method, approx. 5 mg of ground cannabis flowers were weighed into a headspace vial and spiked with 2 μL of ISTD. For calibrators and quality control (QC) samples 10 μL of appropriately diluted standard solutions in methanol were pipetted into the vials and spiked with 2 μL of ISTD. Vials were thermostatted for 20 min at 100°C in agitation mode. Syringe temperature was set to 110°C and inlet temperature was 250°C. A sample volume of 1 mL was applied with a split ratio of 1:1. Samples were measured with a temperature gradient (77°C, hold 2 min, 4°C/min to 135°C, hold 15 min, 4°C/min to 150°C, 27°C/min to 250°C, hold 15 min, flow 1.2 mL/min) and the MS operated in SIM‐scan mode (scan range *m/z* = 40–300). The SIM‐fragments for each analyte are given in Table 1.

#### 2.2.4 Method Validation

Validation was performed in accordance with the guidelines of the German Society of Toxicological and Forensic Chemistry (GTFCh) [62, 63, 64] and included the parameters selectivity, linearity of calibration, analytical limits, accuracy (bias) as well as intra‐ and inter‐day precision. Data were statistically evaluated using Valistat 2.00.1 software (Arvecon; Walldorf, Germany). Selectivity was tested with an empty vial (containing ambient air), a vial spiked with methanol as a blank matrix, vials spiked either solely with commercial terpene mixtures or with ISTD. Since some analytes were present in several of the commercial terpene mixtures, one of the mixtures was selected for validation, in which the analyte was present with ≥ 95% purity in a defined isomer composition, if possible. This was not feasible for *cis/trans*‐β‐ocimene and valencene, as the purities of the reference substances were < 95%. *cis/trans*‐β‐ocimene was calibrated from Terpenes Mega Mixture #1 from Restek with a purity of 93% and valencene was calibrated from Terpene Mixture 1 from Dr. Ehrestorfer with a purity of 88.5% (Table 1). Linearity of calibration was tested by a three‐fold measurement [63] of the calibration series covering up to 11 calibrators in a range between 5 and 1,000 μg/mL. Following the recommendations of Markelow and Guzowski [58], it is convenient to calibrate FET methods to the ‘absolute amount of analyte’ in the vial, which corresponds to 0.05–10 μg, equaling 10–2,000 μg/g referred to a sample weight of 5 mg ground cannabis (Table 2). In the case of *cis/trans*‐β‐ocimene and β‐cedrene, reference substances included in the mixes were mixtures of several isomers with clearly defined percentages in the certificate of analysis (given in Table 1). The actual content of these analytes in the calibration samples was calculated using these percentages (Table 2). Analytical limits were determined in accordance with DIN 32645 [64]. According to the calibration curve method, six evenly distributed calibrators in the range of the expected detection limit were prepared (0.1, 0.5, 1.0, 2.0, 4.0 resp. 7.5 μg/mL corresponding to 0.001, 0.005, 0.01, 0.02, 0.04 resp. 0.075 μg absolute amount of analyte in the vial). Accuracy (bias), intra‐ and inter‐day precision were evaluated by analysing QC samples at low and high concentrations relative to the calibration range (Table 2) in duplicate on eight different days. Acceptance criteria were a bias of ±15% (±20% near the limit of quantification) and a relative standard deviation (RSD) of ≤ 15% (≤ 20% near the limit of quantification) for intra‐ and inter‐day precision.

**TABLE 2:** Calibration models for validated terpenes, including their assignment to the standard mix and the ISTD.

		Cal1	QC_low_	Cal2	Cal3	Cal4	Cal5	Cal6	Cal7	Cal8	QC_high_	Cal9	Cal10	Cal11	ISTD
	μg in vial	0.050	0.0625	0.0750	0.125	0.375	0.500	1.00	1.75	2.50	3.75	5.00	7.50	10.0	
	μg/mL	5.00	6.25	7.50	12.5	37.5	50.0	100	175	250	375	500	750	1,000	
	μg/g (~5 mg sample)	10.0	12.5	15.0	25.0	75.0	100	200	350	500	750	1,000	1,500	2,000	
**No**.	Analyte	Calibration model													
	*Terpenes Mega Mix #1 from Restek*														
1	α‐pinene	Entire range (0.050–10.0 μg)													Decane
2	camphene	Entire range (0.050–10.0 μg)													Decane
3	sabinene	Entire range (0.050–10.0 μg)													Decane
4	β‐myrcene	Entire range (0.050–10.0 μg)													Decane
5	β‐pinene	Entire range (0.050–10.0 μg)													Decane
7	3‐carene	Entire range (0.050–10.0 μg)													Decane
8	α‐terpinene	Entire range (0.050–10.0 μg)													Decane
9	*cis*‐β‐ocimene (28%)	0.0140	0.0175	0.0210	0.0350	0.105	0.140	0.280	0.490	0.700	1.05	1.40	2.10	2.80	Decane
		Entire range (0.0140–2.80 μg)													Decane
10	m‐cymene	Entire range (0.050–10.0 μg)													Decane
11	limonene	Entire range (0.050–10.0 μg)													Decane
12	p‐cymene	Entire range (0.050–10.0 μg)													Decane
13	*trans*‐β‐ocimene (72%)	0.0360	0.0450	0.0540	0.090	0.270	0.360	0.720	1.26	1.80	2.70	3.60	5.40	7.20	Decane
		Entire range, (0.0360–7.20 μg)													Decane
14	eucalyptol	Entire range (0.050–10.0 μg)													Decane
15	o‐cymene	Entire range (0.050–10.0 μg)													Decane
16	γ‐terpinene	Entire range (0.050–10.0 μg)													Decane
17	terpinolene	Entire range (0.050–10.0 μg)													Undecane
18	*trans*‐sabinene hydrate	Low range (0.050–1.00 μg)						High range (1.00–10.0 μg)							Undecane
19	linalool	Low range (0.050–1.00 μg)						High range (1.00–10.0 μg)							Undecane
21	endo‐fenchol	Low range (0.050–1.00 μg)						High range (1.00–10.0 μg)							Undecane
22	isopulegol	Low range (0.050–1.00 μg)						High range (1.00–10.0 μg)							Dodecane
25	terpinene‐4‐ol	Low range (0.050–1.00 μg)						High range (1.00–10.0 μg)							Dodecane
28	α‐terpineol	Low range (0.050–1.00 μg)						Restricted high range (1.00–7.50 μg)						/	Dodecane
30	thymol	Low range (0.050–0.500 μg)					Restricted high range (0.500–7.50 μg)							/	Tridecane
31	carvacrol	Low range (0.050–0.500 μg)					Restricted high range (0.500–7.50 μg)							/	Tridecane
33	*trans*‐β‐farnesene	Entire range (0.050–10.0 μg)													Tetradecane
36	β‐cedrene (20%)	0.010	0.0125	0.0150	0.0250	0.0750	0.100	0.200	0.350	0.500	0.750	1.00	1.50	2.00	Tetradecane
		Entire range (0.010–2.00 μg)													
41	guaiol	Low range (0.050–1.00 μg)						High range (1.00–10.0 μg)							Hexadecane
	*Cannabis Terpene Mix A from Merck*														
20	fenchone	Restricted range (0.05–5.0 μg)											/	/	Undecane
23	camphor	Restricted range (0.05–5.0 μg)											/	/	Dodecane
24	isoborneol	Low range (0.050–1.00 μg)						High range (1.00–10.0 μg)							Dodecane
26	menthol	Low range (0.050–1.00 μg)						High range (1.00–10.0 μg)							Dodecane
29	pulegone	Entire range (0.050–10.0 μg)													Tridecane
32	geranyl acetate	Low range (0.050–1.00 μg)						High range (1.00–10.0 μg)							Tetradecane
34	α‐cedrene	Restricted range (0.050–5.00 μg)											/	/	Tetradecane
37	α‐humulene	Entire range (0.05–10 μg)													Pentadecane
43	α‐cedrol	Low range (0.050–1.75 μg)							High range (1.75–10.0 μg)						Heptadecane
44	α‐bisabolol	Low range (0.050–1.75 μg)							High range (1.75–10.0 μg)						Heptadecane
	*Cannabis Terpene Mix B from Merck*														
27	borneol	Low range (0.05–1.75 μg)							High range (1.75–10.0 μg)						Dodecane
35	β‐caryophyllene	Entire range (0.050–10.0 μg)													Tetradecane
39	*cis*‐nerolidol	Low range (0.050–1.75 μg)							High range (1.75–10.0 μg)						Pentadecane
45	β‐eudesmol	Low range (0.050–1.75 μg)							High range (1.75–10.0 μg)						Heptadecane
	*Terpene Mixture 1 from Dr. Ehrestorfer*														
6	α‐phellandrene	Entire range (0.050–10.0 μg)													Decane
38	valencene	Restricted range (0.050–5.00 μg)											/	/	Pentadecane
42	caryophyllene oxide	Low range (0.05–1.75 μg)							High range (1.75–10.0 μg)						Heptadecane
40	selina‐3‐7‐(11)‐diene	Low range (0.050–1.00 μg)						Restricted high range (1.00–7.50 μg)						/	Hexadexane

#### 2.2.5 Application to Cannabis Flower Material

The method was applied to six different medicinal cannabis strains with similar Δ^9^‐THC content. The selection included two indica/indica‐dominant ‘Kush' strains (Pink Kush: 18.7 wt‐% Δ^9^‐THC, Master Kush: 18.6 wt‐% Δ^9^‐THC), two sativa/sativa‐dominant ‘Haze' strains (Ghost Train Haze: 19.9 wt‐% Δ^9^‐THC, Delahaze: 20.3 wt‐% Δ^9^‐THC) as well as two popular hybrid strains (White Widow: 17.9 wt‐% Δ^9^‐THC, Gorilla Glue 4: 17.8 wt‐% Δ^9^‐THC). In accordance with legal requirements, samples were stored protected from unauthorised access in their original packaging, sealed and protected from light, at room temperature for several months prior to grinding (shelf‐life was expired at the time of measurement). Further information on the medicinal cannabis flowers is given in Table S1. Moreover, terpene profiles of two self‐grown plants of the strain ‘Purple Milkshake’ were analysed. These plants were legally cultivated indoors without any additional artificial measures, air‐dried after harvest and stored in a freezer after grinding. Seeds were purchased from a local shop and Δ^9^‐THC‐contents were determined using a previously validated method [65] (Δ^9^‐THC: Plant #1 15.4 wt‐%, Plant # 2 15.2 wt‐%).

## 3 Results

### 3.1 Method Development

**FIGURE 3:**
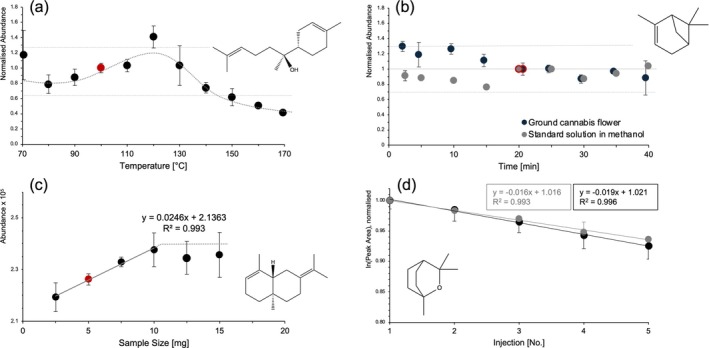
Experimental optimisation of FET. Optimisation of thermostatting temperature using approx. 5 mg of ground cannabis flowers for α‐bisabolol ((a), normalised peak areas to 100°C and sample weight). Optimisation of thermostatting time using approx. 5 mg of ground cannabis flowers or 10 μL of a 100 μg/mL Terpene Mega Mix #1 solution in methanol for α‐pinene ((b), normalised peak areas to weight and 20 min). According to allowed measurement uncertainties in forensic guidelines of ± 30% [66] lines were inserted between a normalised abundance of 0.7–1.3. Sample size optimisation using approx. 2.5–15 mg of ground cannabis flowers for selina‐3,7‐(11)‐diene ((c), absolute peak areas calculated from actual weight to target weight using the rule of three for comparison of duplicates). Verification of full evaporation by multiple headspace extraction (MHE) using 5 mg sample material or 10 μL of a 100 μg/mL Terpene Mega Mix #1 solution in methanol for eucalyptol ((d), logarithmic absolute peak areas). Values represent the mean (*n* = 2) of peak areas, MS‐data was acquired in SIM‐mode.

For the development of the FET, various experiments were carried out to check the condition of full evaporation. At least two representatives of each substance class (monoterpenes, monoterpenoids, sesquiterpenes and sesquiterpenoids) were evaluated. The FET optimisation process is exemplarily illustrated in Figure 3, with further data for each substance class provided in the supplement (Figures S1–S4). Maximum peak areas were reached at slightly higher thermostatting temperatures between 80°C and 120°C in ground cannabis flowers than in standard solutions between 70°C and 90°C (Figures 3a and S1), so that a thermostatting temperature of 100°C was selected as being favourable for all analytes. When gradually increasing the thermostatting time, a constant response was reached after 20 min for ground cannabis flowers and standard solution (Figures 3b and S2) indicating the completion of the evaporation process. Sample amounts between 2.5 and 7.5 mg resulted in a linear increase in peak areas (r^2^ > 0.99), while first saturation effects were observed for higher boiling sesquiterpenes at 10 mg (Figures 3c and S3). Optimised sample quantities of 5 mg showed a linear depletion of peak areas on a logarithmic scale when multiple injections of a single sample were plotted. Slopes were similar to the depletion of signal in standard solutions showing consistently deviations < 30% (±30% as outer limits of the acceptance interval for QC‐samples according to the forensic guideline [66], Figures 3d and S4). Evaluation of full evaporation experiments for alkanes used as ISTDs revealed similar trends to analytes (Figures S1–S8). Although substantially lower peak areas were observed for higher‐boiling alkanes in cannabis samples than in standards (on average up to −78.2%), it was determined that the reduced peak area of ISTDs on cannabis flowers is a reproducible phenomenon (two samples, *n* = 5, standard deviation max. ±12.55%).

**FIGURE 4:**
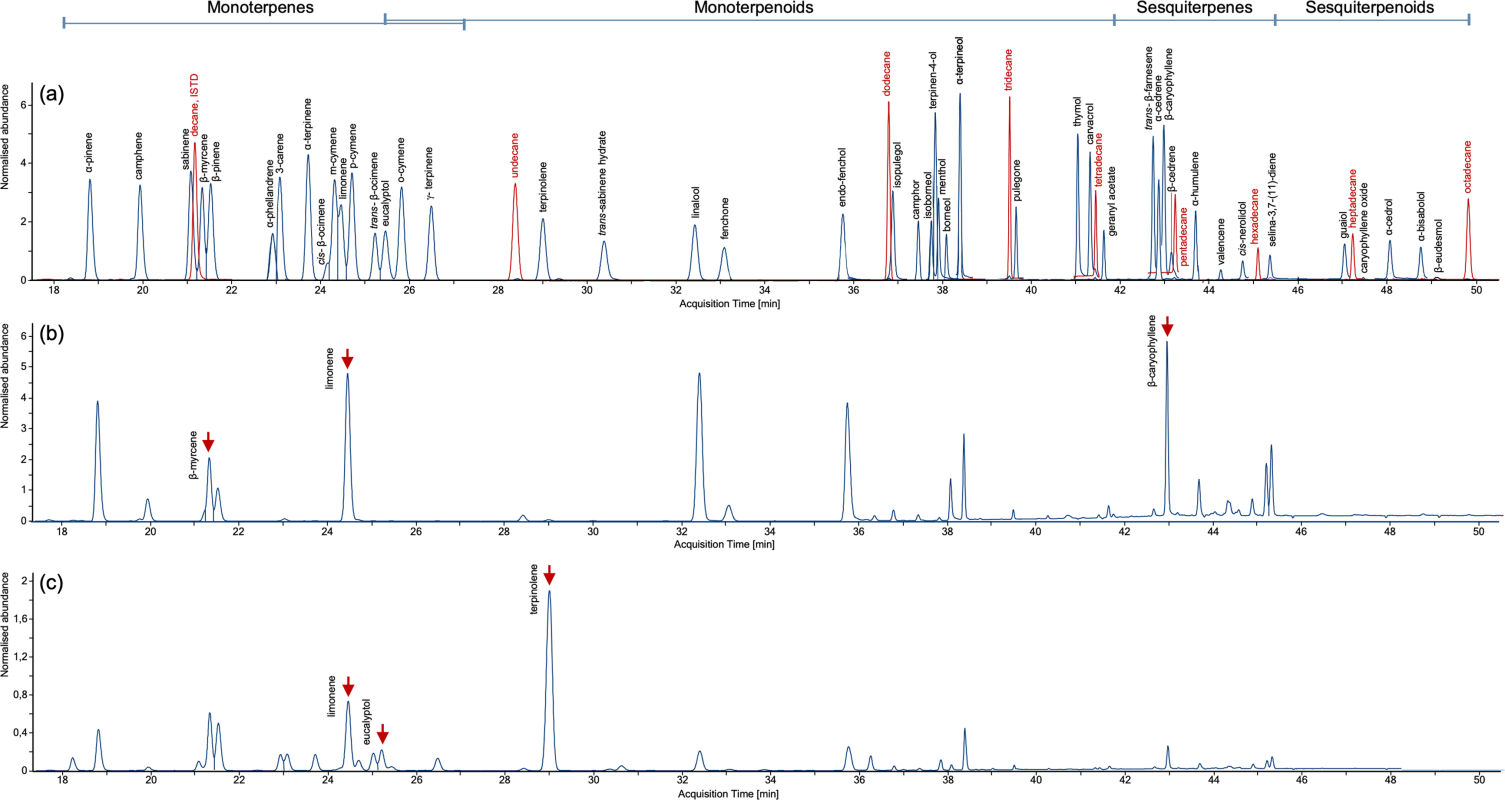
Total ion chromatograms (TIC) of standard solutions and cannabis flower samples. An overlay of different calibration mixtures of calibrator 9 illustrates the whole analyte panel including the RT index mixture as ISTD (a). Differences in the terpene profile are exemplarily depicted by two cannabis samples, strain ‘Master Kush’ (b) and strain ‘Ghost Train Haze’ (c). The three dominant terpenes in the samples according to manufacturer are highlighted.

The isomeric nature of terpenes and their common construction from isoprene subunits leads to similar MS‐fragmentation patterns (Figure S9). Therefore, in terms of selectivity, careful development of the temperature gradient was crucial in order to separate all components sufficiently from each other for an undisturbed quantification within a run time of 53.95 min (cycle time 57.0 min, Figure 4). The elution order of terpenes within the mixes was identified using MS databases (e.g., NIST, Wiley 275) and verified for plausibility using literature data and chromatograms contained in the certificate of analysis. For isomers with very similar spectra (e.g., thymol, carvacrol / m‐cymene, p‐cymene and o‐cymene), the chromatogram obtained was compared with a re‐modelling of the method using available online software (Restek EZGC Chromatogram Modeler, Figure S10). Identifying the elution order of *cis*‐ and *trans*‐β‐ocimene was particularly challenging. While modelling suggested that *trans* elutes before *cis*, collections of retention indices indicate that *cis* elutes before *trans* [67]. The latter matches the proportions that should be contained in the standard mix, as the second peak, presumably *trans*‐β‐ocimene, should be the more dominant one.

### 3.2 Method Validation

**FIGURE 5:**
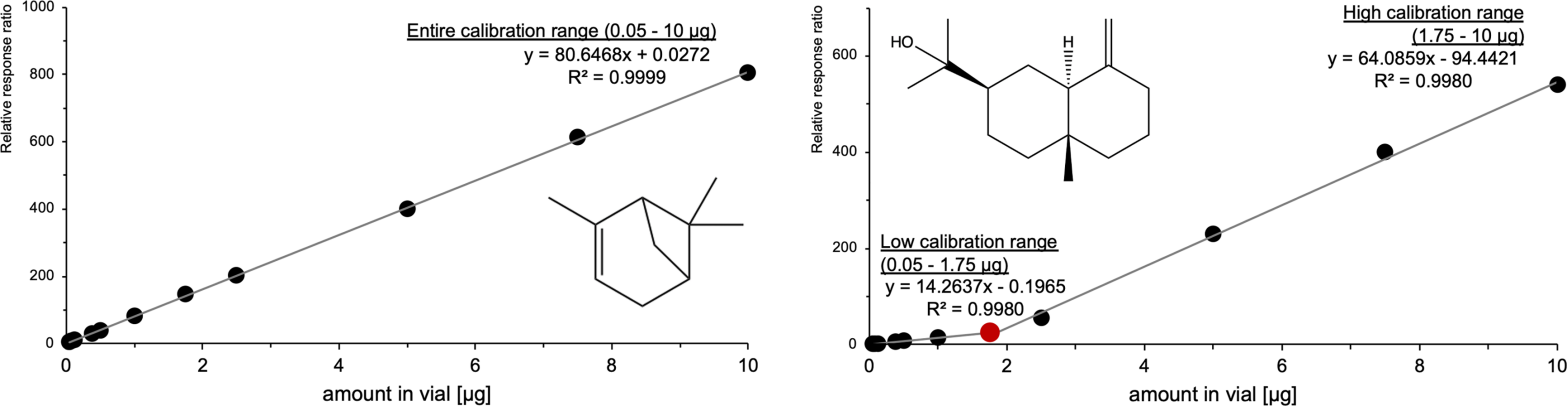
Entire calibration range for first eluting monoterpene α‐pinene and split calibration range for last eluting sesquiterpenoid β‐eudesmol.

No interference of analyte masses by solvent or ISTD mixture could be observed. Also, masses of ISTDs were not interfered by analytes. Terpenes were evaluated with the alkane as ISTD that eluted closest before their retention time. A linear calibration model without weighting could be used for all analytes (variance homogeneity tested by a Cochran test across all concentrations, significance 99%; Mandel linearity test, significance: 99%) with correlation coefficients of > 0.99. For some analytes, however, including most of the monoterpenoids except for eucalyptol, fenchone and camphor, all sesquiterpenoids as well as selina‐3‐7‐(11)‐diene, it was necessary to split the calibration range into two sub‐ranges (Table 2, Figure 5), ensuring that at least 5 calibrators were used per partial calibration [63]. In addition, due to saturation effects, restrictions of calibration ranges were required for fenchone, camphor, α‐terpineol, thymol, carvacrol, α‐cedrene, valencene and selina‐3‐7‐(11)‐diene (Table 2). Analytical limits were consistently below the lowest calibrator. Accuracy (bias), intra‐ and inter‐day precision were within the acceptance criteria for all analytes (Table 3). In addition, it was verified that the reduced peak areas of ISTDs on cannabis flowers (see Section 3.1, Figure S8) do not introduce systematic errors into the quantification by spiking three different cannabis flowers with standard (10 μL of a 100 μg/mL Terpene Mega Mix #1). The absolute quantities determined in the vials fall within a ± 30% range, in line with the acceptance criteria for QCs [66] (Table S2).

**TABLE 3:** Validation results including analytical limits (limit of detection (LOD), limit of quantification (LOQ)), accuracy, intra‐ and inter‐day precision at low and high QC level.

			Analytical limits		Accuracy (bias) % (*n* = 8)		Intra‐day precision % (*n* = 8)		Inter‐day precision % (*n* = 8)	
No.	Analyte	RT [min]	LOD [μg]	LOQ [μg]	Low	High	Low	High	Low	High
1	α‐pinene	18.8	0.00844	0.0292	10.2	0.87	4.7	3.0	6.9	4.9
2	camphene	20.0	0.00758	0.0262	10.0	0.86	3.7	4.9	5.0	5.8
3	sabinene	21.1	0.00762	0.0263	8.3	2.6	4.2	3.7	7.0	4.4
	DECANE	21.2	/	/	/	/	/	/	/	/
4	β‐myrcene	21.4	0.00666	0.0231	7.7	1.9	5.1	4.3	7.5	4.3
5	β‐pinene	21.5	0.00826	0.0286	6.4	1.7	5.0	3.6	8.9	4.7
6	α‐phellandrene	23.0	0.00697	0.0241	10.4	−0.84	2.9	3.0	6.5	5.5
7	3‐carene	23.1	0.00813	0.0281	7.3	−3.7	3.2	6.1	7.4	9.6
8	α‐terpinene	23.7	0.00670	0.0229	8.9	3.8	6.0	3.9	8.1	4.2
9	*cis*‐β‐ocimene	24.2	0.00283	0.00980	9.2	0.38	3.8	5.0	6.8	8.1
10	m‐cymene	24.3	0.00830	0.0287	3.9	3.3	6.6	4.3	11.2	4.3
11	limonene	24.5	0.00689	0.0239	7.8	1.6	5.2	3.8	8.0	4.2
12	p‐cymene	24.7	0.00789	0.0273	3.9	3.8	6.2	3.5	10.8	3.5
13	*trans*‐β‐ocimene	25.2	0.00573	0.0198	7.6	3.8	4.0	4.2	8.2	4.6
14	eucalyptol	25.5	0.00835	0.0289	5.2	−2.0	6.4	5.5	11.0	7.5
15	o‐cymene	25.8	0.00861	0.0290	4.5	1.5	5.2	4.6	10.0	5.0
16	γ‐terpinene	26.5	0.00828	0.0286	6.7	−0.51	6.5	6.3	7.7	7.4
	UNDECANE	28.4	/	/	/	/	/	/	/	/
17	terpinolene	29.0	0.00601	0.0209	6.5	−0.64	5.9	4.9	10.0	4.9
18	*trans*‐sabinene hydrate	30.4	0.00823	0.0284	−6.5	−6.3	7.2	8.5	11.9	8.5
19	linalool	32.4	0.00609	0.0212	4.2	−5.4	7.2	7.4	10.1	7.5
20	fenchone	33.1	0.00731	0.0252	5.2	−4.7	3.5	8.5	5.1	9.4
21	endo‐fenchol	35.8	0.00792	0.0273	2.3	−6.9	7.8	7.0	11.6	7.0
	DODECANE	36.8	/	/	/	/	/	/	/	/
22	isopulegol	36.9	0.00720	0.0249	9.5	−8.5	6.7	6.4	6.7	6.4
23	camphor	37.5	0.00827	0.0286	6.3	−4.2	3.5	8.1	6.3	9.2
24	isoborneol	37.8	0.00839	0.0290	2.6	−4.2	4.2	9.7	10.2	9.8
25	terpinene‐4‐ol	37.8	0.00815	0.0282	−0.5	−5.0	7.7	6.9	11.2	6.9
26	menthol	37.9	0.00827	0.0286	4.0	−5.0	4.2	9.4	5.2	9.4
27	borneol	38.1	0.00789	0.0273	−6.2	−5.5	10.6	8.9	10.6	8.9
28	α‐terpineol	38.4	0.00761	0.0263	6.4	−4.3	4.5	7.2	7.6	7.5
	TRIDECANE	39.5	/	/	/	/	/	/	/	/
29	pulegone	39.7	0.00605	0.0210	8.4	0.78	1.9	8.3	4.6	10.8
30	thymol	41.0	0.00753	0.0260	5.8	−2.0	10.8	9.9	10.8	10.0
31	carvacrol	41.3	0.00719	0.0249	7.0	−1.2	9.5	11.8	9.5	11.8
	TETRADECANE	41.4	/	/	/	/	/	/	/	/
32	geranyl acetate	41.6	0.00824	0.0285	−2.2	−1.3	11.6	12.3	11.6	12.3
33	*trans*‐β‐farnesene	42.7	0.00713	0.0247	3.0	5.0	8.5	4.9	10.6	4.9
34	α‐cedrene	42.9	0.00383	0.0135	−1.5	7.5	3.3	4.4	6.5	7.6
35	β‐caryophyllene	43.0	0.00790	0.0273	−2.4	−2.7	2.8	3.5	13.2	5.0
36	β‐cedrene	43.2	0.00224	0.00783	9.5	−2.1	4.3	11.1	4.9	11.1
	PENTADECANE	43.2	/	/	/	/	/	/	/	/
37	α‐humulene	43.7	0.00293	0.0104	6.9	4.3	1.6	5.1	4.3	6.2
38	valencene	44.3	0.00813	0.0281	7.4	3.0	2.3	4.5	4.5	9.2
39	*cis*‐nerolidol	44.7	0.00786	0.0271	3.9	−1.7	4.0	9.0	7.4	9.5
	HEXADECANE	45.1	/	/	/	/	/	/	/	/
40	selina‐3,7‐(11)‐diene	45.4	0.00839	0.0290	1.8	−2.0	5.0	0.0	7.5	7.2
41	guaiol	47.0	0.00782	0.0270	11.3	−10.0	5.4	4.7	5.4	4.7
	HEPTADECANE	47.2	/	/	/	/	/	/	/	/
42	caryophyllene oxide	47.5	0.00518	0.0181	−5.1	−5.0	6.4	3.6	12.5	8.4
43	α‐cedrol	48.1	0.00608	0.0211	−7.3	−8.5	7.4	4.9	10.6	5.8
44	α‐bisabolol	48.8	0.00818	0.0283	10.6	−9.7	3.5	3.1	5.2	5.1
45	β‐eenudesmol	49.1	0.00668	0.0232	6.2	−3.9	4.9	9.6	5.3	9.6

### 3.3 Application to Cannabis Flower Material

**FIGURE 6:**
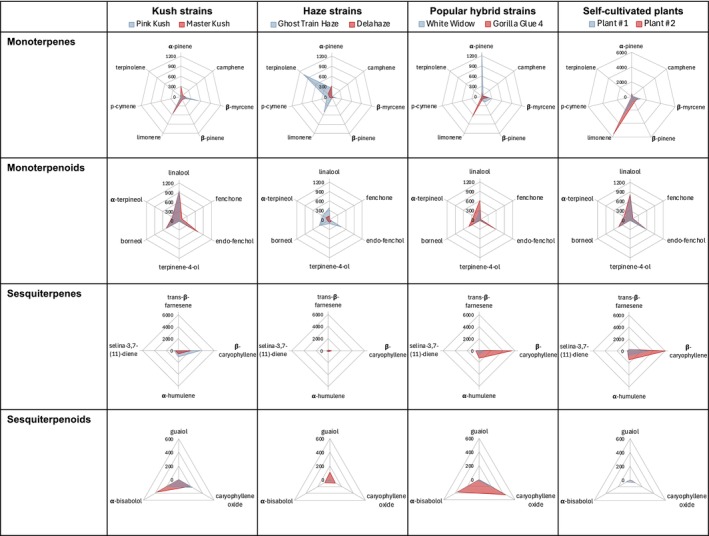
Radar charts of the cannabis strains analysed. To simplify the presentation, only terpenes that were detected in at least one sample at > 100 μg/g were included in the plots. Due to large differences in content, scaling for monoterpenes differs between medicinal cannabis flowers and self‐grown flowers for better representation.

The method was applied to six medicinal cannabis flower samples and two self‐grown plants. Minor interferences in the masses for pulegone, geranyl acetate, and valencene were revealed, although it was clearly evident that the samples were negative due to deviating target/qualifier ratios or missing qualifiers, so that no restrictions in selectivity could be concluded from this. Some terpenes, including m‐cymene, o‐cymene, isopulegol, menthol, pulegone, thymol, carvacrol, geranyl acetate, α‐cedrene, β‐cedrene, valencene, *cis*‐nerolidol and α‐cedrol, were not detected in any of the samples. 3‐carene, p‐cymene, *trans*‐β‐ocimene, eucalyptol, isoborneol, guaiol, α‐bisabolol and β‐eudesmol were only detected in some of the samples. Although sometimes with significant variations in concentrations, α‐pinene, camphene, sabinene, β‐myrcene, β‐pinene, α‐phellandrene, α‐terpinene, *cis*‐β‐ocimene, limonene, γ‐terpinene, terpinolene, *trans*‐sabinene hydrate, linalool, fenchone, endo‐fenchol, camphor, terpinene‐4‐ol, borneol, α‐terpineol, *trans*‐β‐farnesene, β‐caryophyllene, α‐humulene, selina‐3,7‐(11)‐diene and caryophyllene oxide were detected in all of the samples. Sabinene, *cis*‐β‐ocimene, camphor, isoborneol as well as α‐phellandrene, eucalyptol, γ‐terpinene and *trans*‐sabinene hydrate were detected in concentrations of < 10 and < 50 μg/g, respectively. Contrastingly, α‐pinene, β‐myrcene, limonene, terpinolene, β‐caryophyllene and α‐humulene were among the most dominant terpenes, which were also detected in amounts > 1,000 μg/g. In the case of limonene and β‐caryophyllene, the calibration range was exceeded for some samples, so the values were verified by re‐analysing smaller sample weights. For limonene, one sample showed deviations > + 30%, which could indicate saturation effects when extrapolating (Table S3). A detailed overview of the raw data is provided in Table S4. The two chromatograms in Figure 4 provide a first impression of differences in terpene profiles between a ‘Haze’ and a ‘Kush’ strain. To illustrate the differences in more detail, radar charts were created (Figure 6). Longer‐stored medicinal cannabis flowers contained smaller amounts of monoterpenes than self‐grown plants sampled right after harvest and drying, while sesquiterpene and terpenoid levels were comparable. Sativa‐dominant ‘Haze' strains as well as 'White Widow' contained considerably lower amounts of sesquiterpenes than the other samples.

## 4 Discussion

A FET methodology was successfully validated for the analysis of 45 terpenes in cannabis flowers and its application to a set of cannabis strains as real sample material was demonstrated.

### 4.1 Method Development

#### 4.1.1 Derivation of Analyte Selection and Analytical Approach

Due to the differing analytical approaches and discrepancies in terpene profiles reported in the literature, the use of reliable reference standards [52] and the harmonisation of analytical procedures has been recommended [19, 52]. Prior to method development, both of these aspects, analyte selection and choice of an appropriate analytical approach, were carefully considered. Reference standards were almost exclusively bought in the form of commercial terpene mixtures which simplifies the handling of volatile substances. However, the composition of these mixtures, most of which are marketed specifically for terpene analysis in cannabis, has been criticised in the past. While especially relevant sesquiterpenes such as *cis*‐β‐farnesene or β‐selinene are currently missing, rather irrelevant terpenes such as pulegone and menthol with no reports of significant concentrations in cannabis, also not detected at all in this study, are included [52]. Unfortunately, in particular the missing relevant terpenes are usually difficult to obtain as individual reference substances [51, 68] in suitable purity at affordable prices. In this study, a reference substance for selina‐3,7(11)‐diene was purchased separately and added to one of the mixtures, as it is a rather unknown terpene, but has been emphasised in previous studies as one of the predominant sesquiterpenes in cannabis [13, 56, 69]. As no reference substance has been available so far [56], it is not yet sufficiently researched, but could be an interesting marker for forensic purposes. The results confirm that, alongside β‐caryophyllene and α‐humulene, it is one of the dominant sesquiterpenes in cannabis, consistently exceeding levels > 100 μg/g (range 167–683 μg/g). In line with this, a further study reported concentration ranges between 0 and 1330 μg/g (average 249 μg/g) [70]. To be able to at least identify further relevant terpenes that are currently not available as reference substances and possibly optimise the method at a later time, the measurement was carried out in SIM‐scan mode. While quantification was exclusively performed via SIM in accordance with current recommendations [66, 68], unknown terpenes can be matched via the scan‐mode using library workflows. Another problem is the unclear isomeric purity of some commercially available terpene reference substances (e.g. diastereomers with *cis/trans*‐isomerism not properly defined). Therefore, when selecting the terpene mixes used here, attention was paid to ensure that isomeric compositions are comprehensively defined and, when doubts arose, additional information was requested from the manufacturer prior to purchase. Great importance was assigned to clearly presenting the composition of analytes in this study, which is a prerequisite for transparent validation.

As analytical approach, a GC–MS method using the headspace‐based FET was chosen [56, 57]. FET is a very simple technique which does not require tedious sample preparation [71] apart from simply weighing in a small amount of sample in a headspace vial. This is advantageous for the intended use of this method, covering the analysis of medicinal cannabis as well as seized cannabis flowers and making it easier to collect samples at several locations and pass them on to the institute in the form of a pre‐weighed vial. From an analytical point of view, FET is recommended as an effective way to control matrix effects when no clean matrix is available [58, 61]. In general, the term ‘matrix effects’ describes the interference of a quantitative measurement by matrix components of a sample. Strategies to control these effects include the reduction of matrix components and the compensation of their influence in the calibration methodology [72]. The problem of lacking clean matrix has now been recognised and a first commercial product has become available shortly (Zero C, Cayman Chemicals [73]). Approaches for producing surrogate matrix using pre‐heated cannabis flowers for SPME/SHS‐analysis [51] or stripped hops pellets for liquid‐based injection can be found in the literature [55]. For liquid extractions, however, solvents with a wide range of polarities, including methanol [54], ethanol [13, 22, 54], isopropanol [55], butanol [74], ethyl acetate [54], chloroform [43], petroleum ether [38], diethyl ether [75], n‐pentane [44], n‐hexane [74] as well as mixtures of these are currently used. The choice of extraction solvent can influence the extracted terpene profile [74, 75]; for example, nonpolar terpenes (e.g. β‐caryophyllene) are better extracted in nonpolar solvents [76]. In most method developments for terpene analysis, tests of extraction efficiency and, in particular, comparisons of different solvents are either not carried out at all or are described only very briefly without mentioning detailed results [22, 54]. FET overcomes this problem by circumventing any solvent extraction procedures [71]. In headspace sampling, the ‘matrix effect’ is defined as the variability of the partition coefficient for an analyte in different sample matrices [58]. Therefore, the use of stripped matrix in SHS is not recommended since the heating can modify the matrix and thus the partition coefficient [61]. The FET minimises the influence of the partition coefficient by complete evaporation [58]. Hence, from an analytical point of view, FET still appears to be most suitable for controlling matrix effects and capturing an unchanged profile of a cannabis flower [56]. This is finally supported by the fact that FET is currently also recommended as state‐of‐the‐art in application notes of some analytical instrument manufacturers (e.g. Agilent [53, 59], PerkinElmer [77, 78] and Shimadzu [60]).

#### 4.1.2 Experimental Verification of Full Evaporation

As there is no guideline for the development of a FET, the approach used here for optimisation and verification of full evaporation (Figure 2) was derived after extensive literature research: Experiments should be carried out using real sample material, as elongated diffusion pathways in solids (e.g. ground cannabis flowers) may demand higher thermostatting conditions than standard solutions [61]. In order to assess thermal decomposition of analytes and artefact formation under these conditions [79, 80], optimisation tests with standard solutions were nevertheless carried out. Compared to other applications of FET to cannabis flowers (160°C, 15 min [57]; 140°C, 40 min [56]; 140°C, 10 min [78], 120°C, 10 min [59]; 150°C, 30 min [60], 110°C, 10 min [53]), the selected thermostatting conditions (100°C, 20 min) are slightly less harsh. This observation is particularly remarkable, as thermal degradation represented the key limiting factor here and has not been discussed in literature relating to the application of FET to terpenes in cannabis flowers so far. Unfortunately, considerations concerning the derivation of the condition of full evaporation are either not described at all or only very incompletely in these references. While higher‐boiling terpenes and alkanes exhibited maximum peak areas at higher temperatures compared to lower‐boiling ones (e.g. linalool: maximum 90°C in cannabis flowers resp. 80°C in methanolic solution, bp. 198°C; α‐bisabolol 120°C in cannabis flowers resp. 90°C in standard, bp 314°C; ISTD pentadecane: maximum 70°C, bp 270°C; hexadecane 80°C, bp 287°C), they underwent thermal decomposition processes to a considerable extent immediately after reaching the sensitivity maximum when the temperature was further increased. For monoterpenes, thermal decomposition in a closed vessel with ambient air has been described when heated to 120°C [81]. Likewise, thermal treatment of linalool‐dominant essential oil resulted in a significant decrease in linalool content of 31.4% (30 min, 100°C) resp. 70.5% (30 min, 150°C) [82]. Also the unsaturated structure of β‐caryophyllene was described as thermally unstable, prone to autooxidation [76, 83]. For example, after 48 weeks of storage in daylight at room temperature, only 1% of β‐caryophyllene was still detectable, while caryophyllene oxide was the main decomposition product [83]. For hexadecane, used as ISTD, thermal degradation in ambient air was described by autooxidation through radical pathways starting from approx. 122°C [84]. Moreover, terpenes/terpenoids can be converted into each other through thermal decomposition, which can lead to unnoticed biases in the terpene profile, highlighting the importance of examining thermal stability in method development. Terpenes can degrade by dehydrogenation and subsequent aromatisation, oxidative cleavage of C‐C‐double bonds, epoxide formation, allylic oxidation (e.g. β‐pinene ➜ α‐pinene, 3‐carene [85], α‐terpinene ➜ *p*‐cymene, thymol, carvacrol, eucalyptol [81]), whereas terpenoids may decompose by dehydroxylation and cyclisation (e.g. linalool ➜ β‐pinene, *cis/trans*‐ocimene, limonene, terpinolene, α‐terpinene [82]). In the experiments carried out here, no additional new peaks or conspicuous amplifications of existing peaks were observed in the chromatograms at the thermostatting conditions chosen, which would indicate the formation of artefacts. Taken all together, 100°C was chosen as an appropriate compromise to ensure sufficient evaporation of higher‐boiling sesquiterpenoids while reducing the risk of decomposition of thermally less stable monoterpenoids and sesquiterpenes. At this temperature, deviations from the temperature optimum of all individual classes of analytes were within the range of acceptable measurement uncertainties (< 30%) [66], so that the extent of potential systematic error introduced by the choice of temperature appears acceptable.

Since the maximum permittable sample size in FET applications varies depending on analyte [86, 87, 88] and temperature [58], full evaporation needs to be proven with increasing amounts of sample to exclude saturation effects [71, 89, 90, 91, 92]. The chosen sample size of 5 mg ground cannabis is in the same order of magnitude as most of the previously reported data (5 mg [56], 10–50 mg [59], 10–30 mg [60]). A further reference utilises higher sample quantities (100 mg) but also applies the highest thermostatting temperature (160°C) [57]. The amount of solvent used to dispense the standard solutions (10 μL methanol) and ISTDs (2 μL hexane) for calibrators and QCs is also very similar to the total solvent amounts used for spiking in previous applications (e.g. 10 μL ethanol [56] and 20 μL methanol [57]). To ensure reliable quantification, adsorption effects caused by non‐evaporated matrix residues are to be ruled out. If this is the case, logarithmic MHE diagrams of samples and standards should show parallel lines [61, 93]. Significant differences between the calculated slopes can be interpreted as the magnitude of matrix effects [79]. Minor differences in the slopes observed were consistently < 30%, which were regarded as acceptable in terms of permissible measurement deviations.

### 4.2 Method Validation

Although runtimes of approx. 1‐h limit the sample throughput, they are highly recommended for reliable selectivity due to the isomerism of terpenes [52]. In previous terpene analyses of cannabis flowers, a higher number of analytes led to increasing runtimes (e.g. 93 analytes in 73 min [56] and 35 analytes in 35 min [57]). Even if separation of reference substances has been ensured, cannabis contains various further terpenes that can interfere with quantification. Selectivity testing was therefore also evaluated on real sample material, but no major interferences were observed. In terms of sensitivity, discrimination effects have been criticised depending on the analytical approach chosen. Whereas early eluting monoterpenes are preferentially detected by classical SHS (equilibration temperature 70°C [52] resp. 80°C [55]), sesquiterpenes are enhanced after hexane‐solvent extraction [52]. This picture was not reflected in the detection limits of the HS‐FET‐GC/MS method showing similar LODs resp. LOQs throughout the whole analyte panel (at least 0.03 μg as LOQ equalling 6 μg/g in cannabis flowers). Therefore, the method's sensitivity goes even slightly beyond the lowest calibrator (5 μg/mL corresponding to 10 μg/g), which was targeted as the lower limit of the working range. Preliminary considerations regarding a reasonable working range for a forensic marker were based on the low levels typically reported for terpenes in literature [70] or medicinal cannabis batch certificates. Quantification was performed using an ISTD although the use of ISTDs in FET is very inconsistent in literature. For example, cannabis flower samples have already been quantified with [57] and without [56] ISTDs. It has been shown that the precision of a FET method can be improved when using an ISTD. While this was explained to compensate for smaller, possibly production‐related differences in the volumes of HS‐vials [94], ISTD‐normalisation could also be particularly helpful in correcting fluctuations in the device status (e.g. condition of the ion source). During method development, the use of ISTDs was evaluated comprehensively and deuterated terpenes were initially tested (β‐myrcene‐d6, linalool‐d3, α‐terpineol‐d3, α‐bisabolol‐d3, data not shown). At high analyte concentrations, however, these were interfered by undeuterated analogues, resulting in apparent increases in peak areas of ISTDs in higher calibrators. Since identical fragments were often formed due to the low number of deuterium atoms introduced, it was difficult to find masses that were discriminant. Tridecane has been described as a cost‐effective and widely used ISTD for terpene analysis in cannabis flowers after solvent extraction [54, 95]. Moreover, since library comparisons of unknown terpene isomers can be unambiguous due to similar fragmentation patterns of isomers, identification combining MS data with retention times (e.g. via retention index) was recommended [96]. Therefore, the idea to use a linear alkane RT index standard mixture as an ISTD was considered to be beneficial for both quantification and qualification and was successfully implemented. Linearity of calibration (range 5–1,000 μg/mL) was verified for all analytes without weighing, although for some analytes, especially terpenoids, the calibration range needed to be split due to varying slopes or restricted due to saturation. Further full evaporation techniques applied to cannabis covered linear calibration ranges between 0.2 and 100 μg/mL [56], 10 and 1,250 μg/mL [59], resp. two separate linear calibration ranges between 12.5 and 100 μg/mL as well as 78.25 and 2,500 μg/mL [60]. Quadratic calibration models were described for broad calibration ranges (four orders of magnitude) after liquid extraction, which may indicate a concentration‐dependent change in detector response [52]. Since forensic validation guidelines [62, 64] strongly favour the use of linear calibration models — even if statistical tests fail as long as QCs meet the accepted limits — it appears reasonable to split the calibration range in order to still be able to use linear calibration models. However, it should be noted that values in the transition range between the two calibrations may be affected by greater measurement errors. It was emphasised as an advantage of FET that variation in sample weight provides flexibility in the quantified content without the need for re‐calibration [58]. Accordingly, if values above the calibration range were quantified in real sample material, lower sample weights were re‐analysed (Table S3). In particular for monoterpenes, results indicated saturation effects, so that the analysis of adjusted sample weights is to be recommended to verify quantification.

### 4.3 Application to Cannabis Flower Material

Total amounts of quantified terpenes were between 0.264 and 1.83 wt‐% which generally correspond to the order of magnitude of typical terpene content previously reported in literature for cannabis flowers (e.g. 0.5–3.5 wt‐% [56]). Like cannabinoids [97], terpene levels also decrease upon storage. In this study, lower levels of monoterpenes were observed in stored medicinal cannabis flowers than in directly processed self‐grown flowers indicating a predominant loss of the most volatile components. A higher loss of monoterpenes compared to sesquiterpenes during storage (up to 3 months, light‐protected at room temperature) has also been described in early studies, although none of the major components (> 0.1% of total terpene content) completely disappeared within this period [50]. This description corresponds well with the findings here, as the characteristic composition of the terpene profile as well as its dominant components could mostly be confirmed according to the manufacturers' specifications (Table S1). For example, β‐myrcene, β‐caryophyllene and limonene were still among the most dominant terpenes in the two measured indica/indica‐dominant ‘Kush' strains. Comparatively low sesquiterpene contents, combined with high monoterpene contents dominated by terpinolene, could also be detected in both ‘Haze' strains. ‘White Widow’ was characterised by a high α‐pinene and β‐caryophyllene content, which, despite the aged sample material, still closely corresponded to the manufacturer's specifications and usual literature values [13]. In ‘Gorilla Glue 4’, limonene was identified as one of the dominant monoterpenes, although the monoterpene content was significantly reduced, while terpenoid and sesquiterpene contents still met the specifications. In particular, the β‐myrcene content was significantly reduced in both hybrid strains, which may indicate differences in the stability of individual monoterpenes. According to information provided by web shops selling seeds, the strain ‘Purple Milkshake’ is described as hybrid (50% sativa, 50% indica [98] resp. 40% sativa, 60% indica [99]) cross‐bred between ‘Horchata’ and ‘Grandaddy OG’ [98, 100] resp. ‘Purple Punch’ and a special ‘Milkshake OG’‐line [99] with Δ^9^‐THC‐contents ranging from 11–26 wt‐%. Its aroma is described as combining earthy, floral, sweet, and fruity notes with hints of cream and vanilla and a spicy grape scent [99, 100]. Analogous to the strain description, β‐myrcene, linalool, β‐caryophyllene, and limonene were among the dominant terpenes in the profiles of the self‐grown plants. The terpene profile thus allowed a plausibility check of the strain of the self‐grown plants, which would not have been possible solely based on the Δ^9^‐THC content.

## 5 Conclusion

A HS‐FET‐GC/MS analysis was successfully validated for the analysis of 45 terpenes in cannabis flowers. Difficulties during method development included the limited availability of reference substances with sufficient purity and clearly defined isomeric composition. Through measurements in SIM‐scan mode, the methodology was initially designed to be as broad as possible in order to filter out relevant terpenes and, if necessary, optimise the method at a later stage based on this data. The careful choice of a RT index mixture as ISTD not only supports quantification, but also identification of unknown terpenes through the combination of MS data with retention indices. The analytical approach is based upon the FET, a special form of static headspace sampling, and its experimental development was derived in detail. FET offers practical and analytical advantages as it circumvents tedious sample preparation and allows an efficient way to control matrix effects. Surprisingly, thermal degradation of higher‐boiling terpenes turned out to be a key limitation leading to lower optimised thermostatting temperatures than usually described in literature. Thermal decomposition of terpenes has not yet been discussed in the context of analyses of cannabis flowers, although this is highly recommended, as terpenes can convert into each other when exposed to heat, which can lead to unnoticed biases in measured profiles. The exemplary measurement of six medicinal cannabis strains with similar Δ^9^‐THC content revealed differences and similarities in their terpene profiles. Moreover, the identity of the strain ‘Purple Milkshake’ of two self‐grown plants could be verified by determining their terpene profiles. Initial practical data also provided insights into which terpenes are dominant, which are not present at all, and which concentration ranges occur. Measurements on stored medicinal cannabis also highlighted the relevance of further structured stability studies, particularly with regard to monoterpene losses during prolonged storage at room temperature. The methodology is meant to be integrated as a complementary dimension into a workflow for comprehensive profiling of cannabis flowers, including a LC–MS/MS method for cannabinoid analysis [101], and is to be applied to a sample collective of medicinal and seized flowers.

## Conflicts of Interest

The authors declare no conflicts of interest.

## Supporting information


**Table S1:** Additional information on exemplarily examined medicinal cannabis strains.
**Figure S1:** Optimisation of thermostatting temperature using approx. 5 mg of ground cannabis flowers or 10 μL of a 100 μg/mL Terpene Mega Mix #1 solution in methanol. Mean values (*n* = 2) of normalised peak areas (normalised to 100°C, in case of cannabis flowers also normalised to weight) are shown. One example of each substance class (limonene (a); linalool (b); *trans*‐β‐farnesene (c); α‐bisabolol (d)) is presented. MS‐data was acquired in SIM‐mode. According to allowed measurement uncertainties in forensic guidelines of ± 30%^66^ lines were inserted between a normalised abundance of 0.7–1.3.
F**igure S2** Optimisation of thermostatting time using approx. 5 mg of ground cannabis flowers or 10 μL of a 100 μg/mL Terpene Mega Mix #1 solution in methanol. Mean values (*n* = 2) of normalised peak areas (normalised to weight and 20 min) are shown. One example of each substance class (α‐pinene (a); α‐terpineol (b); α‐humulene (c); guaiol (d)) is presented. MS‐data was acquired in SIM‐mode. According to allowed measurement uncertainties in forensic guidelines of ±30%^66^ lines were inserted between a normalised abundance of 0.7–1.3.
**Figure S3:** Sample size optimisation using approx. 2.5–15 mg of ground cannabis flowers. Mean values (*n* = 2) of absolute peak areas (calculated from actual weight to target weight using the rule of three for comparison of duplicates) are shown. One example of each substance class (β‐myrcene (a); fenchone (b); selina‐3,7‐(11)‐diene (c); caryophyllene oxide (d)) is presented. MS‐data was acquired in SIM‐mode.
**Figure S4:** Verification of full evaporation by multiple headspace extraction (MHE) using 5 mg sample material or 10 μL of a 100 μg/mL Terpene Mega Mix #1 solution in methanol. Mean values (*n* = 2) of logarithmic absolute peak areas are shown. One example of each substance class (p‐cymene (a); eucalyptol (b); β‐caryophyllene (c); α‐bisabolol (d)) is presented. MS‐data was acquired in SIM‐mode.
**Figure S5:** Optimisation of thermostatting temperature retention index standard mixture used as ISTDs. Thermostatting optimisation samples of standards were additionally spiked with 2 μL of ISTD. The mean values (*n* = 2) of the normalised peak areas (normalised to 100°C) are shown. MS‐data was acquired in SIM‐mode.
**Figure S6:** Optimisation of thermostatting time: Results for tridecane as an example for an ISTD using approx. 5 mg of ground cannabis flowers or 10 μL of a 100 μg/mL Terpene Mega Mix #1 solution in methanol. The mean values (*n* = 2) of the normalised peak areas (normalised to 20 min) are shown.
**Figure S7:** Sample size optimisation using 1–3 μL ISTD spiked on approx. 5 mg ground cannabis flowers. The mean values (*n* = 2) of the absolute peak areas are shown. MS‐data was acquired in SIM‐mode.
**Figure S8:** Evaluation of ISTDs — Verification of full evaporation by multiple headspace extraction (MHE) using 5 mg sample material or 10 μL of a 100 μg/mL Terpene Mega Mix #1 solution in methanol. Samples were spiked with 2 μL retention index standard mixture. The mean values (*n* = 2) of the logarithmic absolute peak areas (normalised to the first injection of standard solution) are shown. MS‐data was acquired in SIM‐mode. =  
**Figure S9:** MS‐Fragmentation patterns of selected monoterpenes and sesquiterpenes. The mass spectra of the bicyclic monoterpene α‐pinene and the monocyclic monoterpene α‐phellandrene resp. the bicyclic sesquiterpenes of selina‐3,7‐(11)‐diene and valencene show a high level of similarity due to the isomeric nature of terpenes (mass spectra from top to bottom). Mass spectra were recorded in scan‐mode using reference substances.
**Figure S10:** Re‐modelling of chromatographic method using the EZGC‐Modeler by Restek. Sabinene, *trans*‐sabinene hydrate, endo‐fenchol, tepinene‐4‐ol, geranyl acetate, *trans*‐β‐farnesene, α‐cedrene, β‐cedrene, valencene, selina‐3,7‐(11)‐diene, α‐cedrol and β‐eudesmol were not included in the online database, so they could not be considered in the simulation. *Trans*‐nerolidol was included in the model but could not be included in the method due to unclear isomeric composition of standards. In deviation from the model, the elution order of ocimene was assigned in inverse order (*cis*‐β‐ocimene before *trans*‐β‐ocimene), which corresponds to the retention indices in literature and the ratios in Mega Mix #1.
**Table S2:** Verification of terpene quantification by spiking cannabis flowers with standard. Approximately 5 mg of three different cannabis flowers were spiked with 10 μL of a 100 μg/mL Terpene Mega Mix #1 solution each. Target values were calculated based on the terpene content measurements performed previously (calculated using the rule of three based on the exact of the weighed sample) plus the amount of standard added. A representative selection of analytes for each ISTD is shown. Since all analytes for which tridecane was used as ISTD were not detected in the cannabis samples, no results are given for tridecane.
**Table S3:** Quantifications exceeding the calibration range at a sample weight of 5 mg. Quantifications outside the calibration range were verified by weighing smaller quantities.
**Table S4:** Application to cannabis flower material—detailed quantification results and total terpene content. Data measured by weighing quantities less than 5 mg are marked with an (*). Values above 100 μg/g or 50 μg/g are highlighted in colour. Terpenes that were detected at > 100 μg/g in at least one sample and are included in the evaluation in Figure 9 are printed in **bold**. Analytes that were not detected at all or solely < 50 ug/g are shown in grey.

## Data Availability

The data that support the findings of this study is available in the supplementary material of this article.
