# Supplementary material for: HS‐FET‐GC/MS‐Method Development and Validation for Analysis of 45 Terpenes—Creating a Complementary Tool for Comprehensive Profiling of Cannabis Flowers in Forensics
Source: Drug Test Anal. 2025 Nov 20;18(1):118–38. doi: 10.1002/dta.3966 (PMC12796564; doi:10.1002/dta.3966)
Supplement: Supplementary file 1 — Table S1: Additional information on exemplarily examined medicinal cannabis strains. Figure S1: Optimisation of thermostatting temperature using approx. 5 mg of ground cannabis flowers or 10 μL of a 100 μg/mL Terpene Mega Mix #1 solution in methanol. Mean values (n = 2) of normalised peak areas (normalised to 100°C, in case of cannabis flowers also normalised to weight) are shown. One example of each substance class (limonene (a); linalool (b); trans‐β‐farnesene (c); α‐bisabolol (d)) is presented. MS‐data was acquired in SIM‐mode. According to allowed measurement uncertainties in forensic guidelines of ± 30%66 lines were inserted between a normalised abundance of 0.7–1.3. Figure S2 Optimisation of thermostatting time using approx. 5 mg of ground cannabis flowers or 10 μL of a 100 μg/mL Terpene Mega Mix #1 solution in methanol. Mean values (n = 2) of normalised peak areas (normalised to weight and 20 min) are shown. One example of each substance class (α‐pinene (a); α‐terpineol (b); α‐humulene (c); guaiol (d)) is presented. MS‐data was acquired in SIM‐mode. According to allowed measurement uncertainties in forensic guidelines of ±30%66 lines were inserted between a normalised abundance of 0.7–1.3. Figure S3: Sample size optimisation using approx. 2.5–15 mg of ground cannabis flowers. Mean values (n = 2) of absolute peak areas (calculated from actual weight to target weight using the rule of three for comparison of duplicates) are shown. One example of each substance class (β‐myrcene (a); fenchone (b); selina‐3,7‐(11)‐diene (c); caryophyllene oxide (d)) is presented. MS‐data was acquired in SIM‐mode. Figure S4: Verification of full evaporation by multiple headspace extraction (MHE) using 5 mg sample material or 10 μL of a 100 μg/mL Terpene Mega Mix #1 solution in methanol. Mean values (n = 2) of logarithmic absolute peak areas are shown. One example of each substance class (p‐cymene (a); eucalyptol (b); β‐caryophyllene (c); α‐bisabolol (d)) is presented. MS‐data was a [file DTA-18-118-s001.docx]

**HS-FET-GC/MS-Method development and validation for analysis of 45 terpenes – creating a complementary tool for comprehensive profiling**

**of Cannabis flowers in forensics**

**Supplementary material**

Marica Hundertmark, Tanja Germerott, Cora Wunder

Department of Forensic Toxicology, Institute of Legal Medicine, University Medical Center, Mainz, Germany

***Table S1 Additional information on exemplarily examined medicinal cannabis strains.***

| **Cultivar**  (wt-% Δ^9^-THC) | **Information from** | |
| --- | --- | --- |
|  | **medicinal cannabis manufacturer** | **website for recreational cannabis cultivation**  https://www.leafly.com |
| **indica / indica-dominant ‘Kush strains’** | | |
| **Pink Kush**  (18.7 wt-% Δ^9^-THC) | indica-dominant,  limonene 21%,  β-caryophyllene 16 %,  β-myrcene 16 %,  nerolidol 14 %,  linalool 13 %,  α-bisabolol 6%,  α-humulene 5 %  (percentages relative to the total terpene content, average over several batches) | also called ‘Pink OG’, indica-dominant hybrid with  β-myrcene,  limonene and  β-caryophyllene  as dominant terpenes  https://www.leafly.com/strains/pink-kush |
| **Master Kush**  (18.6 wt-% Δ^9^-THC) | β-caryophyllene 58 %,  limonene 21 %,  β-myrcene 21 %  (ratio of the three highest concentrated terpenes to each other) | also called ‘High Rise’, ‘Grandmaster Kush’, and ‘Purple SoCal Master Kush’, indica with  β-myrcene,  β-caryophyllene  and limonene  as dominant terpenes,  bred in Netherlands https://www.leafly.com/strains/master-kush |
| **sativa / sativa-dominant ‘Haze strains’** | | |
| **Ghost Train Haze**  (19.9 wt-% Δ^9^-THC) | sativa-dominant,  38 % terpinolene,  15 % eucalyptol,  10 % limonene,  8 % β-pinene,  6 % β-myrcene,  4 % α-pinene,  3 % linalool  (percentages relative to the total terpene content, average over several batches) | sativa with  terpinolene,  limonene and  β-myrcene  as dominant terpenes,  US-American cross-bred  of ‘Ghost OG’ and ‘Neville’s wrack’  https://www.leafly.com/strains/ghost-train-haze |
| **Delahaze**  (20.3 wt-% Δ^9^-THC) | sativa-dominant,  32 % terpinolene,  14 % ocimene,  12 % β-myrcene,  11 % β-caryophyllene,  9 % nerolidol,  6 % α-bisabolol,  16 % further terpenes  (percentages relative to the total terpene content, average over several batches) | 70 % sativa,  terpinolene,  β-myrcene and  pinene  as dominant terpenes,  cross-bred between  ‘Mango Haze’ and ‘Lemon Skunk’  from Netherlands  https://www.leafly.com/strains/delahaze |
| **popular hybrid strains** | | |
| **White Widow**  (17.9 wt-% Δ^9^-THC) | Quantitative terpene profile given, **three dominant terpenes**:  **1.66 mg/g α-pinene,**  0.06 mg/g camphene,  0.84 β-pinene,  **2.30 mg/g β-myrcene,**  0.03 mg/g 3-carene,  < 0.03 mg/g α-terpinene,  < 0.03 mg/g p-cymene,  0.45 mg/g limonene,  < 0.03 mg/g ocimene,  0.06 mg/g γ-terpinene,  0.08 mg/g terpinolene,  0.30 mg/g linalool,  < 0.03 mg/g isopulegol,  0.26 mg/g geraniol,  **1.46 mg/g β-caryophyllene,**  0.70 mg/g α-humulene,  0.29 mg/g nerolidol,  <0.03 mg/g guaiol,  0.12 mg/g α-bisabolol | balanced hybrid of approx.  60 % sativa, 40 % indica,  β-myrcene,  β-caryophyllene and  pinene  as dominant terpenes,  one of the world’s most famous strains, first bred in 1990s in the Netherlands  https://www.leafly.com/strains/white-widow |
| **Gorilla Glue 4**  (17.8 wt-% Δ^9^-THC) | Quantitative terpene profile given, **three dominant terpenes**:  0.498 mg/g α-pinene,  0.069 mg/g camphene,  0.655 mg/g β-pinene,  **5.408 mg/g β-myrcene,**  1.914 mg/g limonene,  0.611 mg/g ocimene,  0.861 mg/g terpinolene,  0.686 mg/g linalool,  0.046 mg/g geraniol,  **7.756 mg/g β-caryophyllene,**  **2.387 mg/g α-humulene,**  0.107 mg/g nerolidol,  0.784 mg/g α-bisabolol,  0.347 mg/g α-terpinolene | also called ‘Glue’, ‘Original Glue’, hybrid  with 37 % sativa and 63 % indica,  β-caryophyllene,  β-myrcene and  limonene  as dominant terpenes,  US-american cross-bred between Chem’s Sister, Sour Dubb, and Chocolate Diesel  https://www.leafly.com/strains/original-glue |

***Figure S1 Optimisation of thermostatting temperature using approx. 5 mg of ground cannabis flowers or 10 µL of a 100 µg/mL Terpene Mega Mix #1 solution in methanol.*** *Mean values (n = 2) of normalised peak areas (normalised to 100 °C, in case of cannabis flowers also normalised to weight) are shown. One example of each substance class (limonene (a); linalool (b); trans-β-farnesene (c); α-bisabolol (d)) is presented. MS-data was acquired in SIM-mode. According to allowed measurement uncertainties in forensic guidelines of ± 30 %^66^ lines were inserted between a normalised abundance of 0.7 – 1.3.*

***Figure S2 Optimisation of thermostatting time using approx. 5 mg of ground cannabis flowers or 10 µL of a 100 µg/mL Terpene Mega Mix #1 solution in methanol.*** *Mean values (n = 2) of normalised peak areas (normalised to weight and 20 min) are shown. One example of each substance class (α-pinene (a); α-terpineol (b); α-humulene (c); guaiol (d)) is presented. MS-data was acquired in SIM-mode. According to allowed measurement uncertainties in forensic guidelines of ± 30 %^66^ lines were inserted between a normalised abundance of 0.7 – 1.3.*


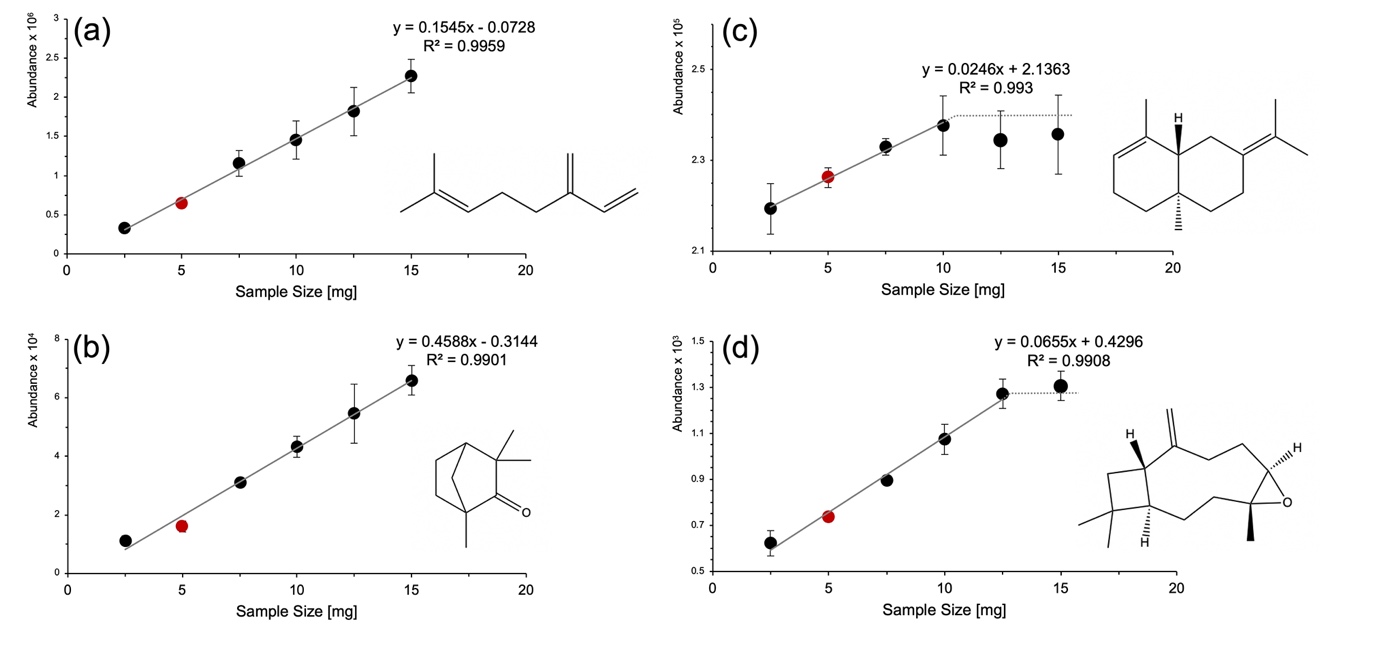


***Figure S3 Sample size optimisation using approx. 2.5 – 15 mg of ground cannabis flowers.*** *Mean values (n = 2) of absolute peak areas (calculated from actual weight to target weight using the rule of three for comparison of duplicates) are shown. One example of each substance class (β-myrcene (a); fenchone (b); selina-3,7-(11)-diene (c); caryophyllene oxide (d)) is presented. MS-data was acquired in SIM-mode.*

***Figure S4 Verification of full evaporation by multiple headspace extraction (MHE) using 5 mg sample material* *or 10 µL of a 100 µg/mL Terpene Mega Mix #1 solution in methanol*.** *Mean values (n = 2) of logarithmic absolute peak areas are shown. One example of each substance class (p-cymene (a); eucalyptol (b); β-caryophyllene (c); α-bisabolol (d)) is presented. MS-data was acquired in SIM-mode.*


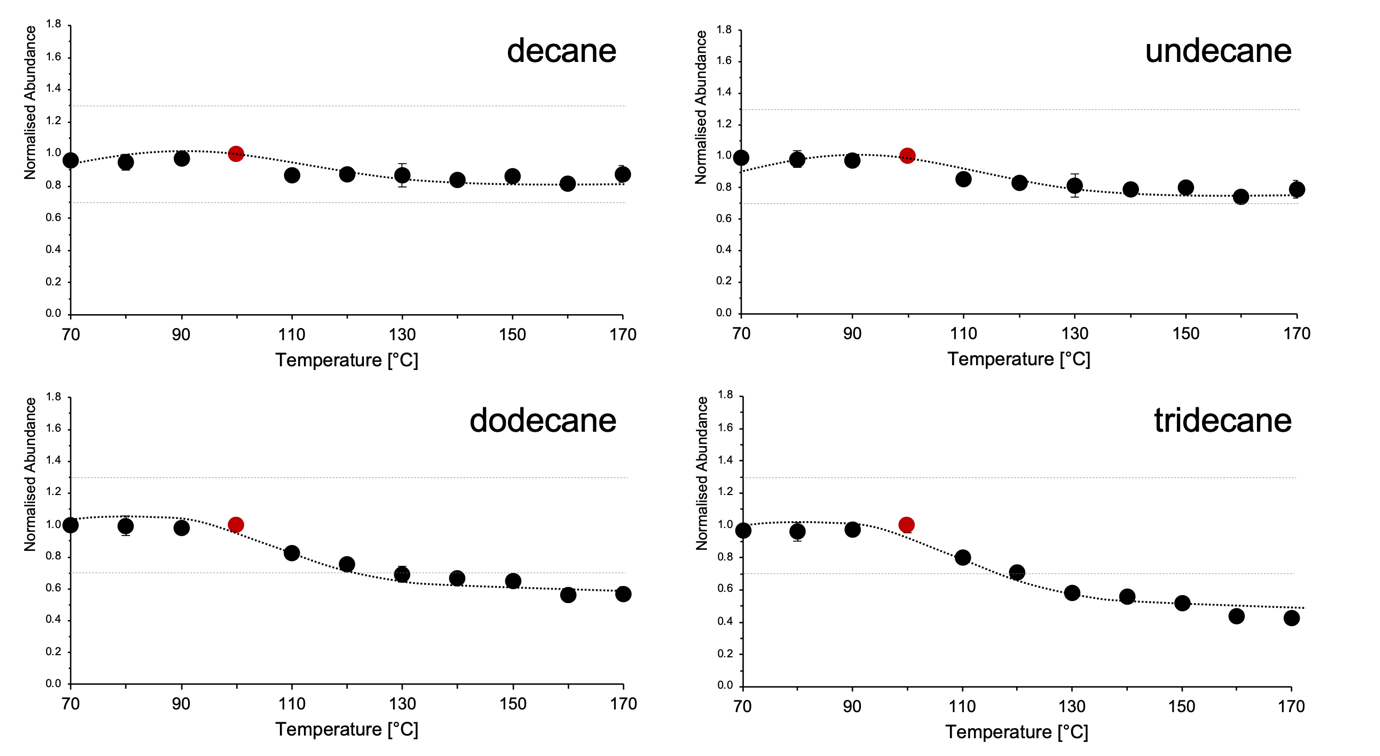

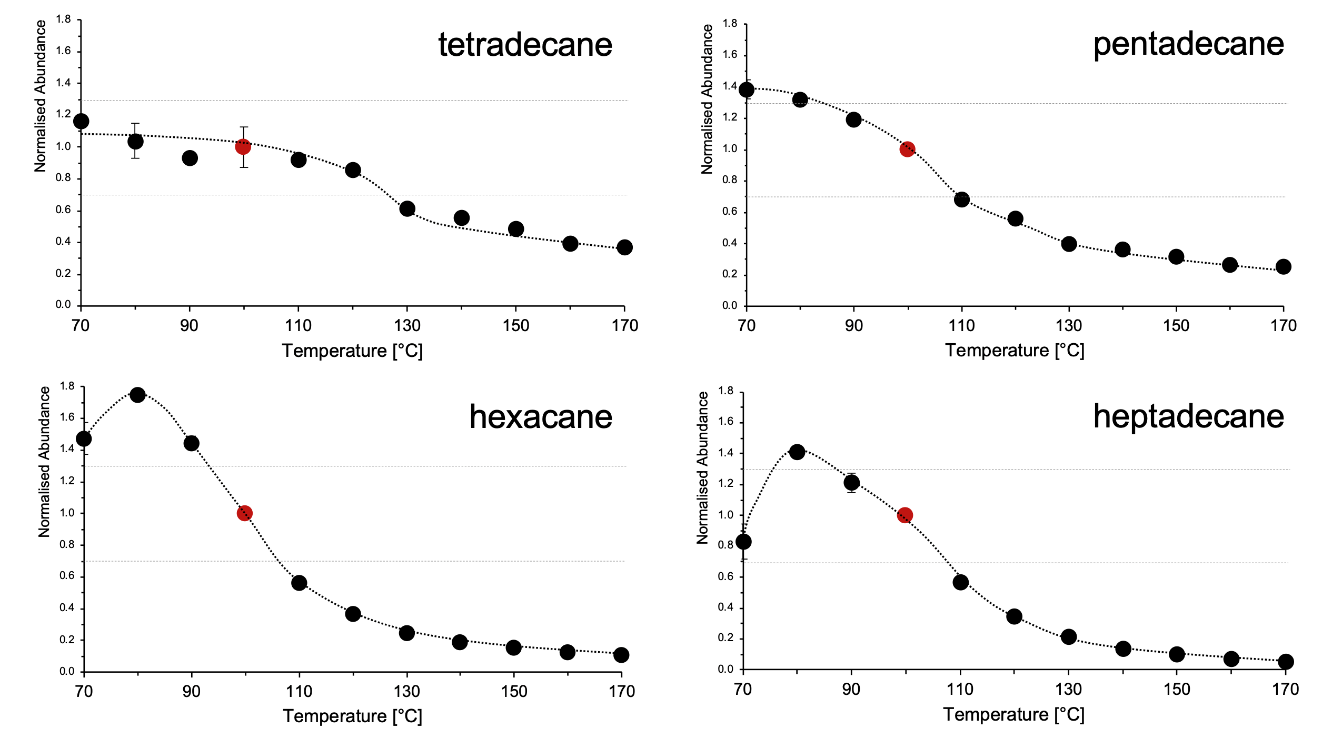


***Figure S5 Optimisation of thermostatting temperature retention index standard mixture used as ISTDs.*** *Thermostatting optimisation samples of standards were additionally spiked with 2 µL of ISTD. The mean values (n = 2) of the normalised peak areas (normalised to 100 °C) are shown. MS-data was acquired in SIM-mode.*

***Figure S6 Optimisation of thermostatting time: Results for tridecane as an example for an ISTD using approx. 5 mg of ground cannabis flowers or 10 µL of a 100 µg/mL Terpene Mega Mix #1 solution in methanol.*** *The mean values (n = 2) of the normalised peak areas (normalised to 20 min) are shown.*


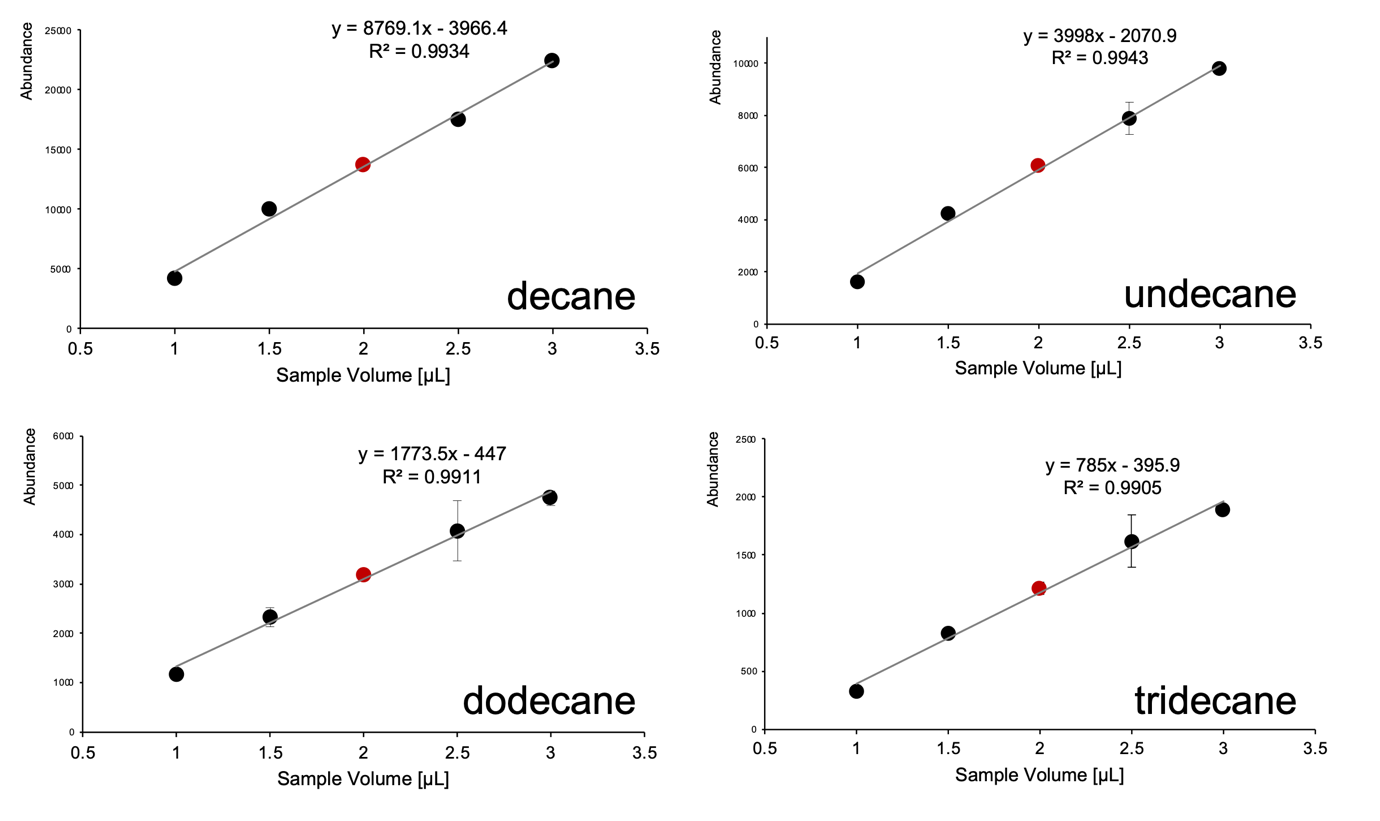

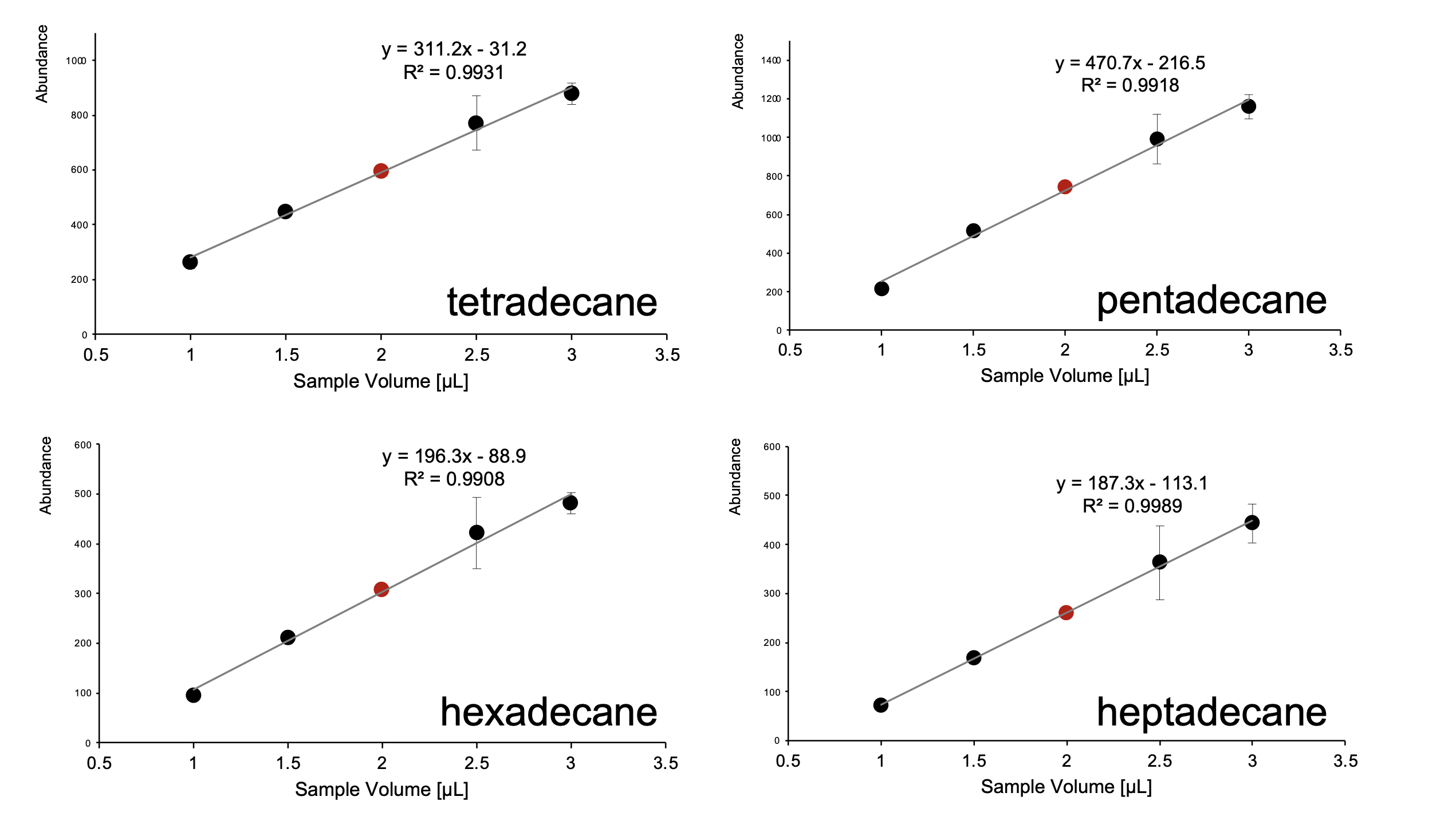


***Figure S7 Sample size optimisation using 1 – 3 µL ISTD spiked on approx. 5 mg ground cannabis flowers.*** *The mean values (n = 2) of the absolute peak areas are shown. MS-data was acquired in SIM-mode.*


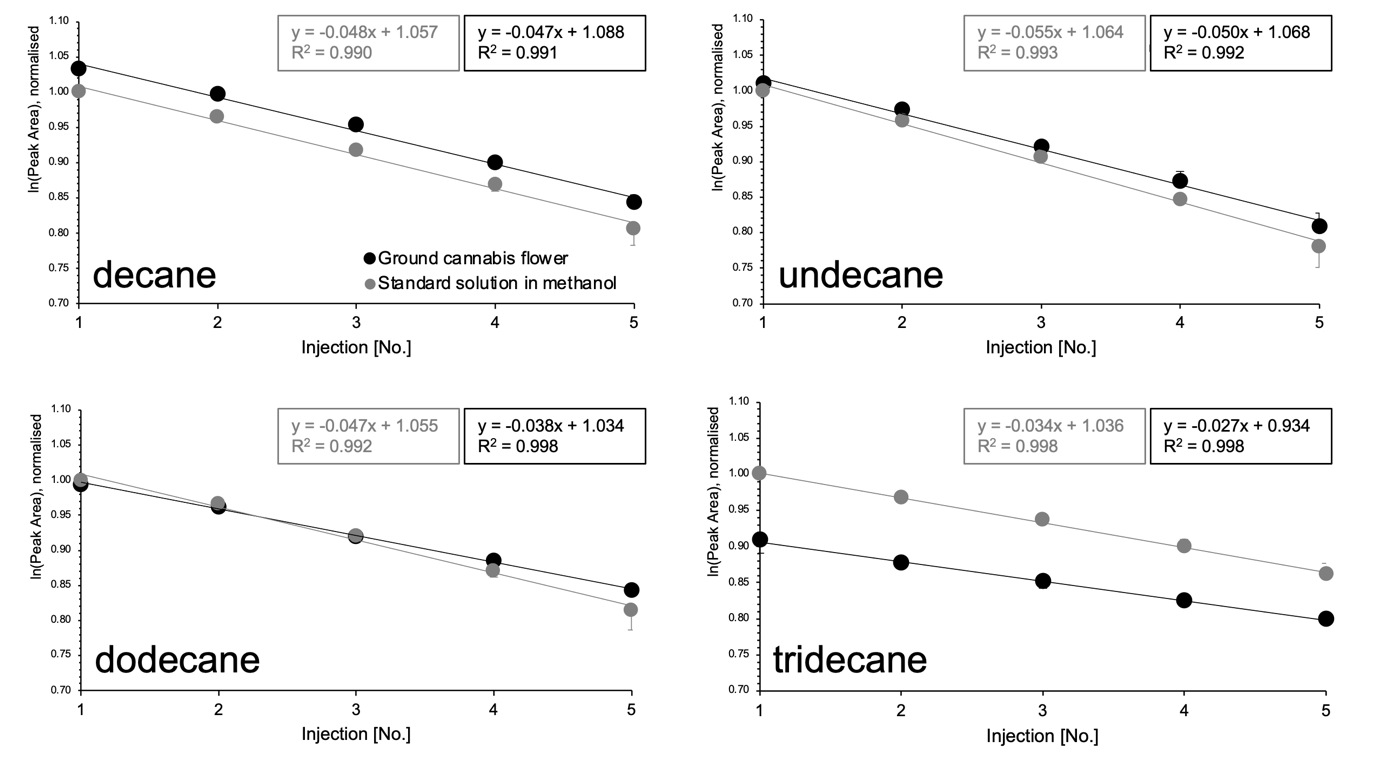

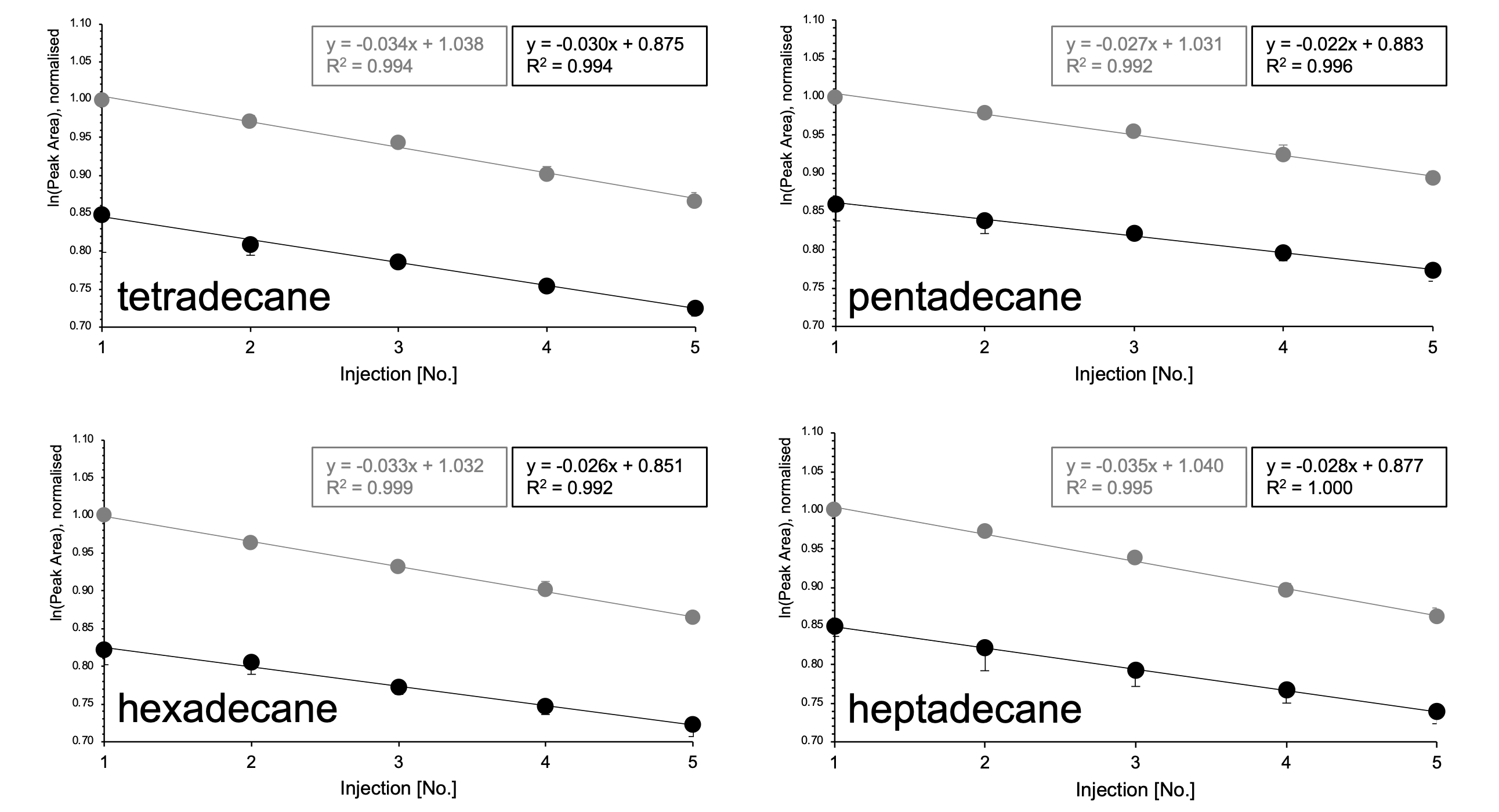


***Figure S8 Evaluation of ISTDs – Verification of full evaporation by multiple headspace extraction (MHE) using 5 mg sample material or 10 µL of a 100 µg/mL Terpene Mega Mix #1 solution in methanol.*** *Samples were spiked with 2 µL retention index standard mixture. The mean values (n = 2) of the logarithmic absolute peak areas (normalised to the first injection of standard solution) are shown. MS-data was acquired in SIM-mode.*

***Figure S9 MS-Fragmentation patterns of selected monoterpenes and sesquiterpenes.*** The mass spectra of the bicyclic monoterpene α-pinene and the monocyclic monoterpene α-phellandrene resp. the bicyclic sesquiterpenes of selina-3,7-(11)-diene and valencene show a high level of similarity due to the isomeric nature of terpenes (mass spectra from top to bottom). Mass spectra were recorded in scan-mode using reference substances.


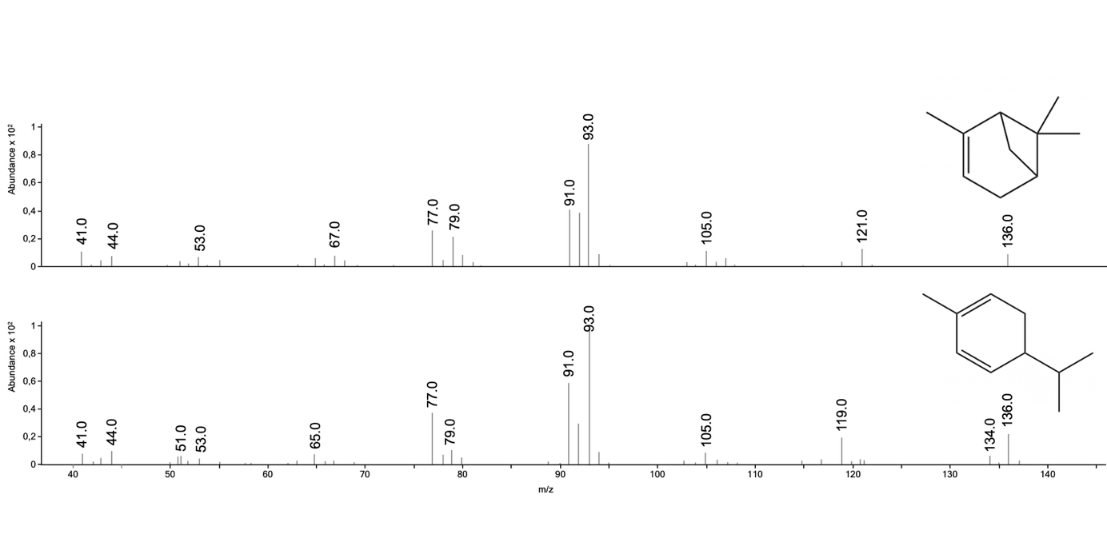

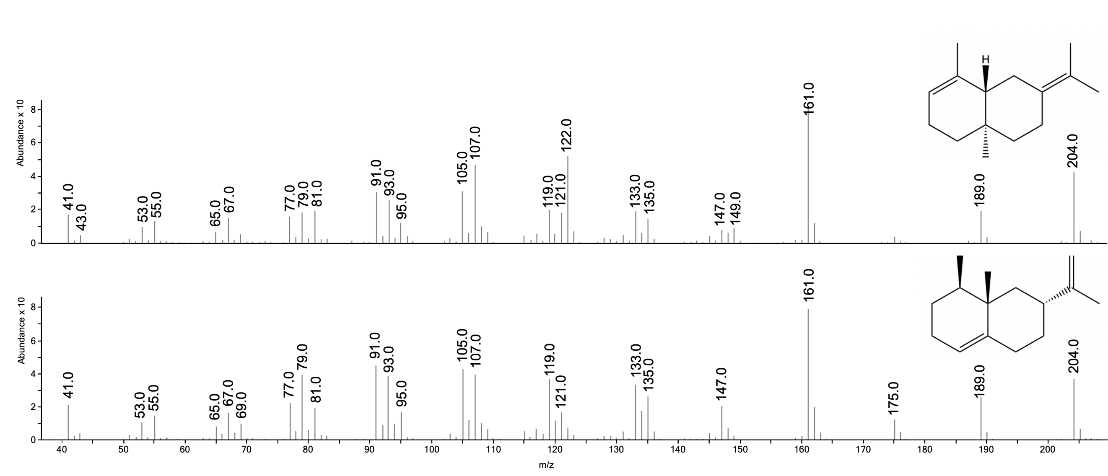

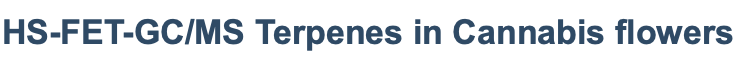

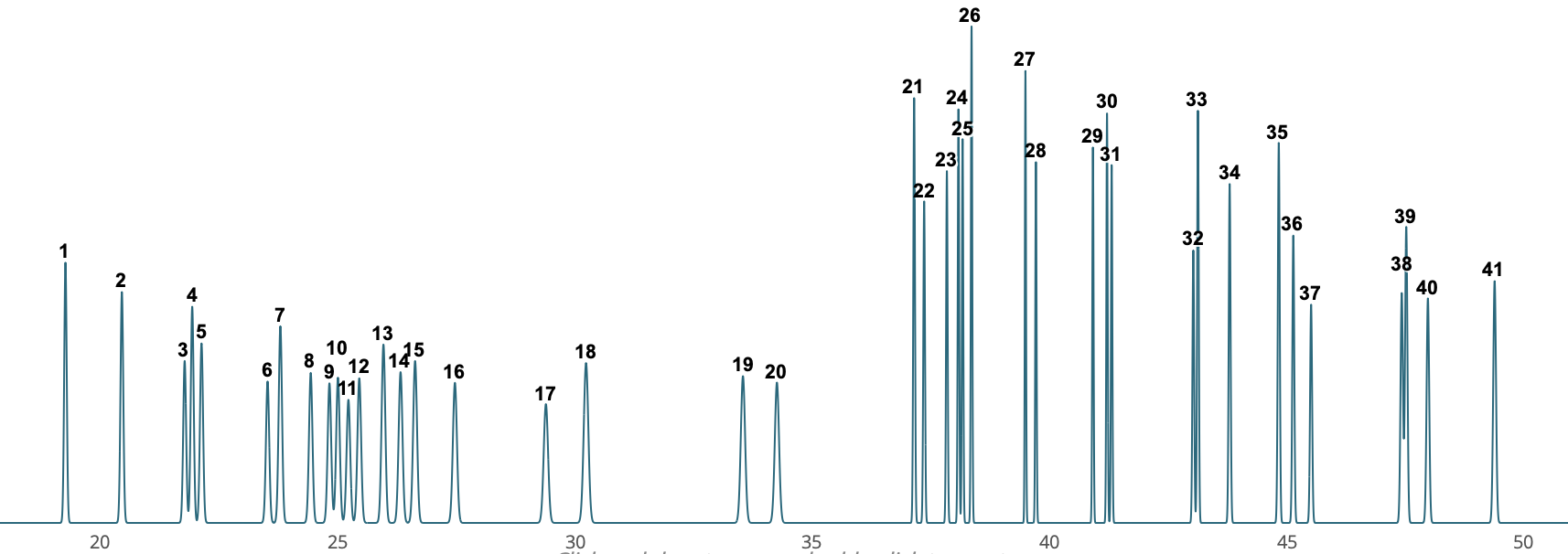

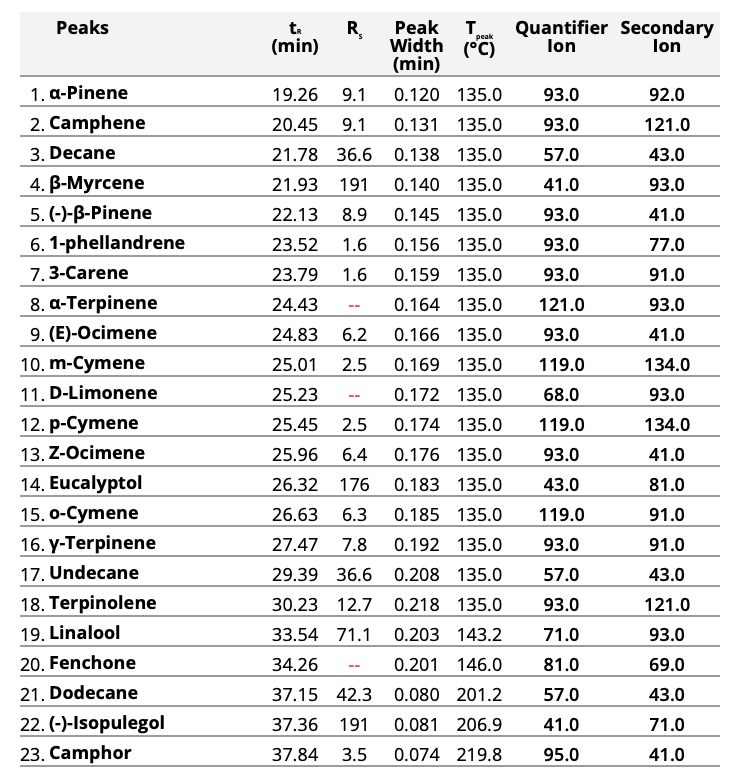

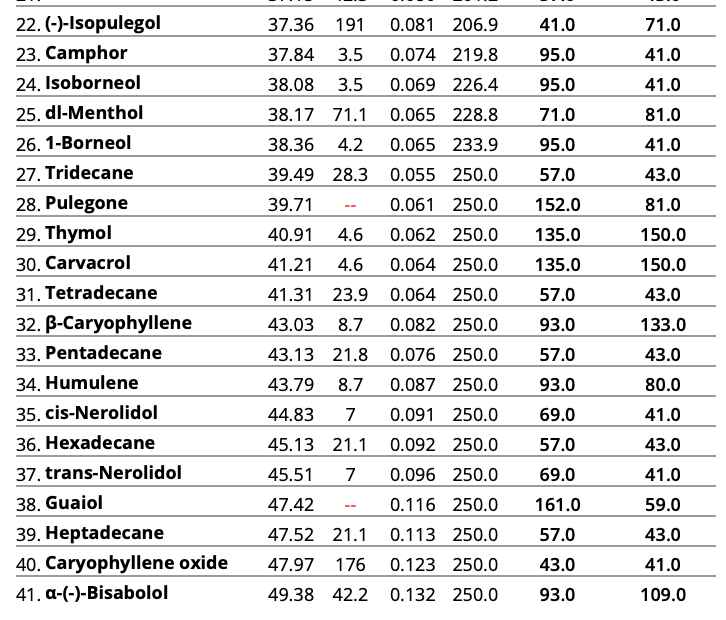

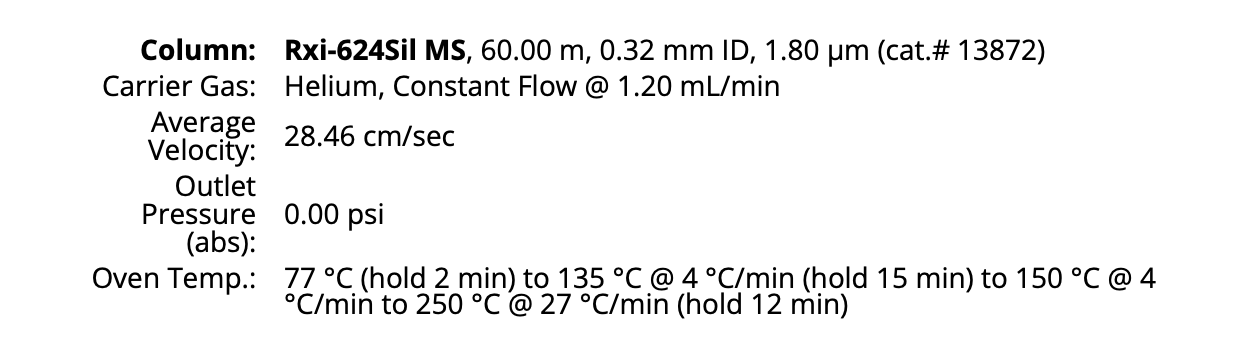


***Figure S10 Re-modelling of chromatographic method using the EZGC-Modeler by Restek.*** *Sabinene, trans-sabinene hydrate, endo-fenchol, tepinene-4-ol, geranyl acetate, trans-β-farnesene, α-cedrene, β-cedrene, valencene, selina-3,7-(11)-diene, α-cedrol and β-eudesmol were not included in the online database, so they could not be considered in the simulation. Trans-nerolidol was included in the model but could not be included in the method due to unclear isomeric composition of standards. In deviation from the model, the elution order of ocimene was assigned in inverse order (cis-β-ocimene before trans-β-ocimene), which corresponds to the retention indices in literature and the ratios in Mega Mix #1.*

***Table S2 Verification of terpene quantification by spiking cannabis flowers with standard. Approximately 5 mg of three different cannabis flowers were spiked with 10 µL of a 100 µg/mL Terpene Mega Mix #1 solution each.*** *Target values were calculated based on the terpene content measurements performed previously (calculated using the rule of three based on the exact of the weighed sample) plus the amount of standard added. A representative selection of analytes for each ISTD is shown. Since all analytes for which tridecane was used as ISTD were not detected in the cannabis samples, no results are given for tridecane.*

| **Analyte** | **Ghost Train Haze** [5.6 mg] | | | **Delahaze** [4.8 mg] | | | **Gorilla Glue 4** [5.4 mg] | | |
| --- | --- | --- | --- | --- | --- | --- | --- | --- | --- |
|  | **Absolute amount of analyte** [µg in vial] | | | | | | | | |
|  | **Target value** | **Actual value** | **Deviation** [%] | **Target value** | **Actual value** | **Deviation** [%] | **Target value** | **Actual value** | **Deviation** [%] |
| Decane as ISTD | | | | | | | | | |
| α-pinene | 2.40 | 2.87 | + 19.5 | 2.57 | 3.24 | + 25.8 | 1.65 | 1.66 | + 1.01 |
| β-myrcene | 1.81 | 2.08 | + 14.6 | 1.19 | 1.47 | + 23.2 | 2.04 | 1.79 | - 12.4 |
| Undecane as ISTD | | | | | | | | | |
| terpinolene | 6.94 | 8.40 | + 21.0 | 1.66 | 2.10 | + 26.3 | 1.03 | 1.22 | + 18.3 |
| linalool | 3.19 | 2.34 | - 26.7 | 1.64 | 1.47 | - 9.96 | 4.32 | 3.25 | - 24.9 |
| Dodecane as ISTD | | | | | | | | | |
| terpinene-4-ol | 1.64 | 1.21 | - 25.9 | 1.37 | 1.16 | - 15.0 | 1.08 | 1.29 | + 19.72 |
| α-terpineol | 2.41 | 2.11 | - 12.6 | 1.64 | 1.19 | - 27.8 | 2.12 | 2.26 | + 2.05 |
| Tetradecane as ISTD | | | | | | | | | |
| *trans*-β-farnesene | 1.06 | 1.21 | + 14.1 | 1.41 | 1.80 | + 27.5 | 1.09 | 1.30 | + 20.1 |
| α-humulene | 2.15 | 1.95 | - 8.95 | 1.92 | 1.92 | + 0.34 | 8.08 | 8.95 | + 10.8 |
| Hexadecane as ISTD | | | | | | | | | |
| guaiol | 1.62 | 1.15 | - 29.2 | / | / | / | / | / | / |
| Heptadecane as ISTD | | | | | | | | | |
| α-bisabolol | 1.23 | 1.26 | + 2.03 | 1.41 | 1.83 | + 29.3 | 3.00 | 2.69 | - 10.3 |

***Table S3 Quantifications exceeding the calibration range at a sample weight of 5 mg.***

*Quantifications outside the calibration range were verified by weighing smaller quantities.*

| **Sample** | **Weight**  [mg] | **Terpene content** [µg/g] | **Deviation**  [%] |
| --- | --- | --- | --- |
| **limonene** | | | |
| Purple Milkshake, Plant #1 | 5.7 | 2100 | + 24.6 |
|  | 1.5 | 2610 |  |
| Purple Milkshake, Plant #2 | 5.1 | 3530 | + 58.6 |
|  | 1.6 | 5600 |  |
| **β-caryophyllene** | | | |
| Pink Kush | 5.0 | 3770 | - 20.8 |
|  | 2.1 | 2990 |  |
| Master Kush | 5.3 | 2140 | + 2.20 |
|  | 2.9 | 2180 |  |
| Gorilla Glue 4 | 5.5 | 5560 | - 10.4 |
|  | 1.3 | 4990 |  |
| Purple Milkshake, Plant #1 | 5.7 | 3320 | - 3.64 |
|  | 1.5 | 3200 |  |
| Purple Milkshake, Plant #2 | 5.7 | 6120 | + 4.85 |
|  | 1.6 | 6420 |  |

***Table S4 Application to cannabis flower material – detailed quantification results and total terpene content.*** *Data measured by weighing quantities less than 5 mg are marked with an (*). Values above 100 µg/g or 50 µg/g are highlighted in colour. Terpenes that were detected at > 100 µg/g in at least one sample and are included in the evaluation in Figure 9 are printed in* ***bold****. Analytes that were not detected at all or solely < 50 ug/g are shown in grey.*

| **No.** | **Analyte** | **Terpene content** [µg/g] | | | | | | | |
| --- | --- | --- | --- | --- | --- | --- | --- | --- | --- |
|  |  | **Medicinal cannabis strains** | | | | | | **Self-cultivated** | |
|  |  | **Kush strains** | | **Haze strains** | | **Popular hybrid strains** | | **Purple Milkshake** | |
|  |  | **Pink Kush** | **Master Kush** | **Ghost Train Haze** | **Delahaze** | **White Widow** | **Gorilla Glue 4** | **Plant #1** | **Plant #2** |
| **1** | **α-pinene** | **98.7** | **314** | **251** | **328** | **1270** | **120** | **334** | **468** |
| **2** | **camphene** | **18.3** | **64.5** | **18.1** | **7.73** | **46.8** | **22.9** | **105** | **130** |
| 3 | sabinene | <LOQ | <LOQ | <LOQ | 6.20 | <LOQ | <LOQ | <LOQ | <LOQ |
| **4** | **β-myrcene** | **519** | **190** | **144** | **40.4** | **283** | **192** | **1230** | **854** |
| **5** | **β-pinene** | **45.2** | **93.3** | **29.2** | **36.0** | **182** | **61.6** | **592** | **801** |
| 6 | α-phellandrene | <LOQ | 7.87 | 34.4 | 12.6 | 8.96 | 7.80 | 84.6 | 9.63 |
| 7 | 3-carene | nd | nd | 66.0 | 18.2 | nd | nd | nd | nd |
| 8 | α-terpinene | <LOQ | <LOQ | 54.0 | 12.3 | <LOQ | <LOQ | <LOQ | <LOQ |
| 9 | *cis*-β-ocimene | 2.24 | 2.06 | 6.18 | 2.14 | 2.36 | 1.91 | 5.02 | 11.1 |
| 10 | m-cymene | nd | nd | nd | nd | nd | nd | nd | nd |
| **11** | **limonene** | **485** | **563** | **506** | **39.7** | **96.8** | **655** | **2610*** | **5600*** |
| **12** | **p-cymene** | **<LOQ** | **<LOQ** | **151** | **68.9** | **<LOQ** | **nd** | **<LOQ** | **nd** |
| 13 | *trans*-β-ocimene | 4.10 | 3.77 | 71.7 | 13.4 | <LOQ | nd | 3.58 | 5.43 |
| 14 | eucalyptol | nd | nd | 48.8 | 34.1 | 15.1 | <LOQ | nd | nd |
| 15 | o-cymene | nd | nd | nd | nd | nd | nd | nd | nd |
| 16 | γ-terpinene | <LOQ | <LOQ | 40.7 | 13.7 | <LOQ | <LOQ | <LOQ | <LOQ |
| **17** | **terpinolene** | **17.6** | **9.45** | **1060** | **137** | **7.55** | **6.25** | **25.0** | **61.6** |
| 18 | sabinene hydrate | 9.08 | 8.32 | 30.6 | 35.8 | 10.3 | 11.9 | 26.1 | 32.3 |
| **19** | **linalool** | **937** | **954** | **391** | **133** | **333** | **615** | **735** | **800** |
| **20** | **fenchone** | **41.5** | **103** | **5.22** | **20.2** | **15.2** | **24.4** | **83.7** | **92.4** |
| **21** | **fenchol** | **452** | **712** | **427** | **68.1** | **296** | **571** | **551** | **557** |
| 22 | isopulegol | nd | nd | nd | nd | nd | nd | nd | nd |
| 23 | camphor | <LOQ | 6.17 | <LOQ | <LOQ | <LOQ | <LOQ | <LOQ | 6.22 |
| 24 | isoborneol | <LOQ | 6.28 | <LOQ | nd | <LOQ | 7.25 | 7.47 | 9.24 |
| **25** | **terpinene-4-ol** | **15.4** | **15.8** | **113** | **76.2** | **9.98** | **14.3** | **11.4** | **13.6** |
| 26 | menthol | nd | nd | nd | nd | nd | nd | nd | nd |
| **27** | **borneol** | **435** | **481** | **372** | **56.9** | **206** | **405** | **393** | **425** |
| **28** | **α-terpineol** | **232** | **243** | **252** | **134** | **105** | **225** | **175** | **220** |
| 29 | pulegone | nd | nd | nd | nd | nd | nd | nd | nd |
| 30 | thymol | nd | nd | nd | nd | nd | nd | nd | nd |
| 31 | carvacrol | nd | nd | nd | nd | nd | nd | nd | nd |
| 32 | geranyl acetate | nd | nd | nd | nd | nd | nd | nd | nd |
| **33** | ***trans*-β-farnesene** | **14.0** | **9.49** | **11.0** | **85.7** | **49.3** | **16.0** | **251** | **302** |
| 34 | α-cedrene | nd | nd | nd | nd | nd | nd | nd | nd |
| **35** | **β-caryophyllene** | **3770** | **2140** | **548** | **575** | **835** | **5560** | **3320** | **6120** |
| 36 | β-cedrene | nd | nd | nd | nd | nd | nd | nd | nd |
| **37** | **α-humulene** | **1070** | **559** | **205** | **192** | **627** | **1310** | **694** | **1470** |
| 38 | valencene | nd | nd | nd | nd | nd | nd | nd | nd |
| 39 | *cis*-nerolidol | nd | nd | nd | nd | nd | nd | nd | nd |
| **40** | **selina-3,7-(11)-diene** | **683** | **522** | **297** | **167** | **204** | **537** | **327** | **279** |
| **41** | **guaiol** | **nd** | **nd** | **nd** | **107** | **nd** | **nd** | **nd** | **nd** |
| **42** | **caryophyllene oxide** | **231** | **197** | **35.3** | **96.6** | **246** | **444** | **84.9** | **54.2** |
| 43 | α-cedrol | nd | nd | nd | nd | nd | nd | nd | nd |
| **44** | **α-bisabolol** | **194** | **367** | **41.6** | **86.1** | **25.4** | **370** | **62.0** | **nd** |
| 45 | β-eudesmol | nd | nd | nd | 53.1 | 11.9 | nd | nd | nd |
| **Total monoterpenes** | | **1190** | **1250** | **2430** | **737** | **1900** | **1070** | **4990** | **7950** |
| **Total sesquiterpenes** | | **5520** | **3210** | **1050** | **935** | **1670** | **7410** | **4340** | **7870** |
| **Total terpenoids** | | **2560** | **3100** | **1740** | **971** | **1320** | **2700** | **2380** | **2510** |
| **Total terpene content** [µg/g] | | **9270** | **7570** | **5230** | **2640** | **4890** | **11200** | **11700** | **18300** |
| **Total terpene content** [wt-%] | | **0.972** | **0.757** | **0.523** | **0.264** | **0.489** | **1.12** | **1.17** | **1.83** |
